# Fc Gamma Receptor CD64 Modulates the Inhibitory Activity of Infliximab

**DOI:** 10.1371/journal.pone.0043361

**Published:** 2012-08-24

**Authors:** Kacper A. Wojtal, Gerhard Rogler, Michael Scharl, Luc Biedermann, Pascal Frei, Michael Fried, Achim Weber, Jyrki J. Eloranta, Gerd A. Kullak-Ublick, Stephan R. Vavricka

**Affiliations:** 1 Division of Gastroenterology and Hepatology, University Hospital Zurich, Zürich, Switzerland; 2 of behalf of the SwissIBD Cohort Study, Zürich, Switzerland; 3 Department of Pathology, Institute for Surgical Pathology, University Hospital Zurich, Zürich, Switzerland; 4 Department of Clinical Pharmacology and Toxicology, University Hospital Zurich, Zürich, Switzerland; 5 Division of Gastroenterology and Hepatology, Hospital Triemli, Zürich, Switzerland; University of Leuven, Rega Institute, Belgium

## Abstract

**Background:**

Tumor necrosis factor (TNF) is an important cytokine in the pathogenesis of inflammatory bowel disease (IBD). Anti-TNF antibodies have been successfully implemented in IBD therapy, however their efficacies differ among IBD patients. Here we investigate the influence of CD64 Fc receptor on the inhibitory activity of anti-TNFs in cells of intestinal wall.

**Methods:**

Intestinal cell lines, monocytes/macrophages and peripheral blood mononuclear cells (PBMCs) were used as models. The efficacies of adalimumab, infliximab and certolizumab-pegol were assessed by RT-PCR for target genes. Protein levels and localizations were examined by Western blotting and immunofluorescence. Antibody fragments were obtained by proteolytic digestion, immunoprecipitation and protein chip analysis. Knock-down of specific gene expression was performed using siRNAs.

**Results:**

Infliximab had limited efficacy towards soluble TNF in cell types expressing Fc gamma receptor CD64. Both adalimumab and infliximab had lower efficacies in PBMCs of IBD patients, which express elevated levels of CD64. Infliximab-TNF complexes were more potent in activating CD64 in THP-1 cells than adalimumab, which was accompanied by distinct phospho-tyrosine signals. Blocking Fc parts and isolation of Fab fragments of infliximab improved its efficacy. IFN-γ-induced expression of CD64 correlated with a loss of efficacy of infliximab, whereas reduction of CD64 expression by either siRNA or PMA treatment improved inhibitory activity of this drug. Colonic mRNA expression levels of CD64 and other Fc gamma receptors were significantly increased in the inflamed tissues of infliximab non-responders.

**Conclusions:**

CD64 modulates the efficacy of infliximab both *in vitro* and *ex vivo*, whereas the presence of this receptor has no impact on the inhibitory activity of certolizumab-pegol, which lacks Fc fragment. These data could be helpful in both predicting and evaluating the outcome of anti-TNF therapy in IBD patients with elevated systemic and local levels of Fc receptors.

## Introduction

Inflammatory bowel disease (IBD) is a chronic, relapsing/remitting inflammatory condition of the gastrointestinal tract, which can be subdivided into two types: Crohn's disease (CD) and ulcerative colitis (UC). Although many pro-inflammatory cytokines have been implicated in the pathology of IBD, one of them, namely tumor necrosis factor (TNF), plays a pivotal role in the course of the disease [1]. TNF is synthesized as a 26 kDa precursor, known as membrane-bound TNF (mTNF). Soluble TNF is produced by proteolysis of mTNF mediated by TNF-alpha converting enzyme (TACE), which resides at the plasma membrane [2]. Both soluble and membrane TNFs can bind to either of the two TNF receptors (TNFR1 or TNFR2), triggering a cascade of intracellular signaling leading to the activation of two major transcription factors involved in pro-inflammatory response: NF-κB and AP-1. Consequently, TNF-stimulated cells produce and secrete a subset of pro-inflammatory cytokines, such as IL-1β, IL-8 and TNF, which further relay the cascade of inflammation in surrounding extracellular environment [3]. Additionally, the effects of TNF include induction of apoptosis, fibrosis, and migration of immune cells.

Based on the crucial role of TNF in inflammation, it was assumed that anti-TNF therapy would be potentially beneficial in chronic inflammatory conditions such as IBD [4] or rheumatic diseases [5]. Currently, there are three anti-TNF therapeutics approved for clinical use: chimeric mouse/human monoclonal anti-TNF IgG_1_ antibody (infliximab; IFX), humanized monoclonal anti-TNF IgG_1_ antibody (adalimumab; ADA) and pegylated humanized anti-TNF Fab′ fragment (certolizumab-pegol; CZP). Until now, numerous *in vitro* models have been employed in order to study the efficacy of these drugs. Most of those studies focus on the comparison between different anti-TNFs using single type of assays or overexpression systems. However, what is lacking so far is the comparison between different cell types potentially targeted by TNF at the site of inflammation. In addition to the classical TNF neutralizing effect, anti-TNF agents are also capable of inducing mTNF-dependent signaling [6]–[8], complement-dependent cytotoxicity (CDC), antibody-dependent cellular cytotoxicity (ADCC) and induction of apoptosis in monocytes [9]–[11]. It has been reported that all three drugs exhibit nearly similar binding affinities towards TNF [12]. The outcome of anti-TNF therapy may also result from other molecular mechanisms, such as inhibition of apoptosis [13]. It may be that at the sites of inflammation several different mechanisms operate simultaneously.

Interestingly, it has been reported that anti-TNF therapeutics bind to Fc receptors in an Fc fragment-dependent manner [14]. In line with these findings, it has been recently demonstrated that anti-TNF agents modulate regulatory functions of immune cells via their Fc region [15] and that IFX can induce wound healing by activating regulatory macrophages [16]. However, on one hand, these studies lack an insight into functional consequences of these drugs for neutralizing soluble TNF, and on the other, did not investigate the involvement of other cell types important for the pathophysiology of IBD. Until now, there are no reports describing consequences of activation of Fc receptors and their downstream signaling by anti-TNF therapeutics, despite the fact that such interactions have been implicated as an important component of the immunological and therapeutic responses [17]–[19].

Here, we report that binding of infliximab to CD64 modulates its inhibitory activity in different cell types of intestinal wall and that this has consequences for the infliximab therapy outcome in IBD patients.

## Results

### Infliximab exhibits limited inhibitory capacity in blocking TNF-mediated inflammatory responses in cells expressing low and high affinity Fc receptors

**Figure 1:**
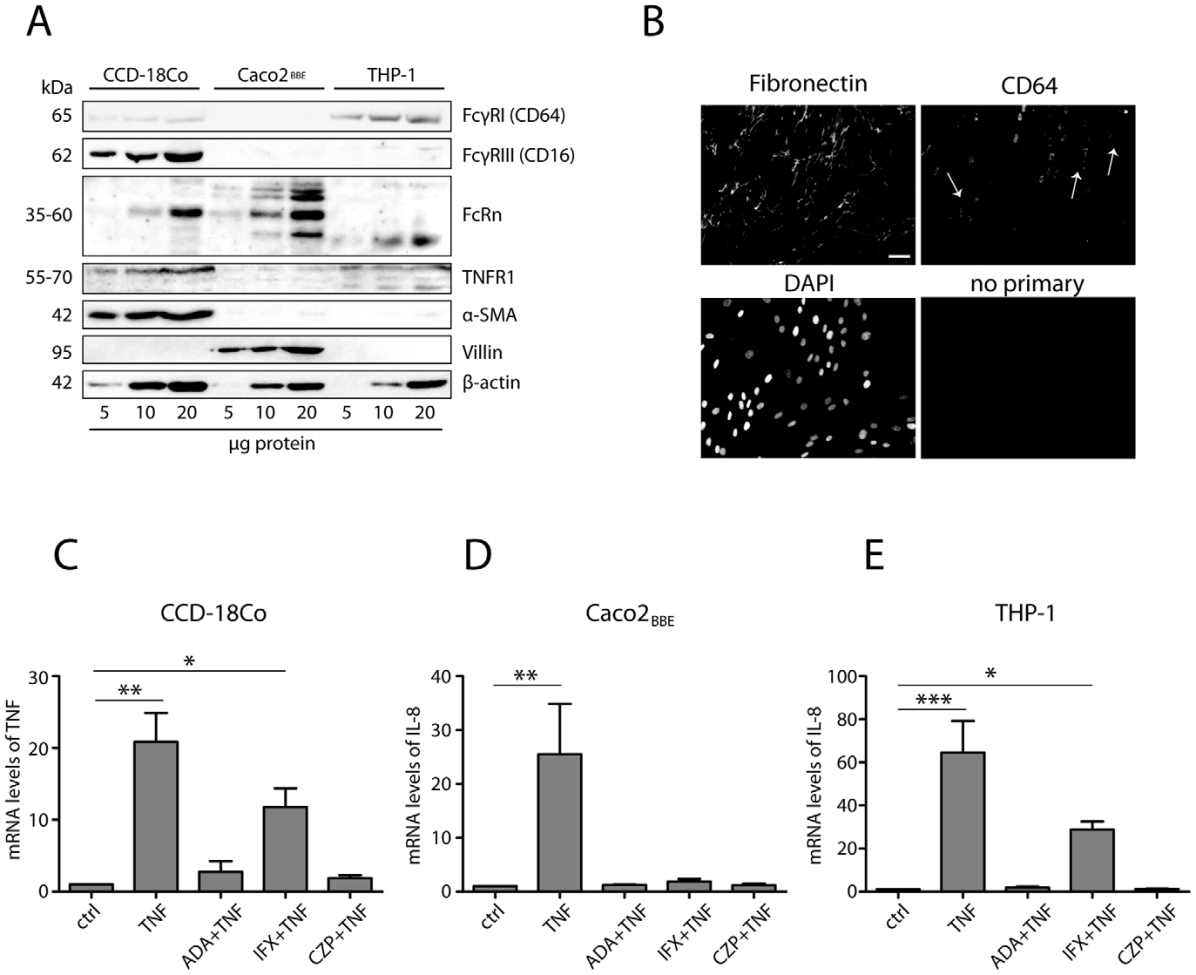
The presence of Fc receptors has a negative effect on the inhibitory efficacy of infliximab. Different cell types composing intestinal wall were examined for the presence of Fc gamma receptors. Villin and α-SMA were used to confirm cell type-specific phenotypes (A). Immunofluorescence analysis of CD64-expressing CCD-18Co fibroblasts. Cells expressing CD64 at the plasma membrane are indicated by arrows. Size bar: 50 µm (B). Intestinal fibroblasts CCD-18Co (C), intestinal epithelial cells Caco2*_BBE_* (D) and THP-1 cells (E) were pre-incubated with anti-TNF therapeutics, and subsequently treated with human recombinant TNF to induce pro-inflammatory response. Values on Y axis represent mRNA expression levels relative to ß-actin. Columns represent mean values of four independent experiments measured in triplicate. Error bars represent SD. *p<0.05; **p<0.01; ***p<0.001.

To test whether the inhibitory efficacy of anti-TNF therapeutics towards soluble TNF depends on the presence of Fc receptors, we first screened different cell types of intestinal wall for the presence of Fc receptors. Both intestine-derived fibroblasts and monocytes/macrophages expressed detectable amounts of CD64 and CD16 (Figure 1A). Neonatal Fc receptor (FcRn) was detected only in epithelial cells and fibroblasts. Because the expression of Fc receptors in fibroblasts is induced upon human cytomegalovirus (CMV) infection [20], we tested intestinal fibroblasts for the presence of viral DNA polymerase. As expected, both cell lines were CMV-positive, as indicated by the specific PCR product (Figure S1). Consistent with immunoblots, we detected CD64 in the nuclear envelope and on the cell surface (Figure 1B, arrows), which is in agreement with previously published observations [21]. Before testing the inhibitory efficacy of IFX and other anti-TNFs, we determined optimal conditions for the soluble TNF-mediated inflammatory responses in the cell lines under study (Figure S2). All responses were specific regarding both signaling pathways and transcription factor activation (Figure S3). After optimizing experimental conditions, we tested the inhibitory efficacy of anti-TNF therapeutic in cell culture. Interestingly, in intestinal fibroblasts, IFX had limited inhibitory efficiency, whereas both ADA and CZP fully blocked TNF-mediated response (Figure 1C). All three drugs efficiently prevented soluble TNF-mediated response in intestinal epithelial cell line Caco2*_BBE_* (Figure 1D), which expressed neither CD16 nor CD64. A similar correlation between limited inhibitory capacity of IFX and the presence of CD64 was observed in THP-1 cells (Figure 1E). In concert with these findings, we observed that in fibroblastic cell lines from CD fistulizing patients (F188, F148 and F141), which express high amounts of both CD64 and CD16, IFX also had limited efficacy (Figure S4). Limited inhibitory efficacy towards soluble TNF was also observed when another humanized anti-TNF IgG1, golimumab, was tested in both fibroblasts and monocytic THP-1 cells, but consistently with previous observations for other anti-TNFs, not in Caco2*_BBE_* cells (Figure S5).

### Anti-TNF therapeutic antibodies have limited efficacy in blocking soluble TNF-mediated responses in peripheral blood mononuclear cells (PBMCs)

**Figure 2:**
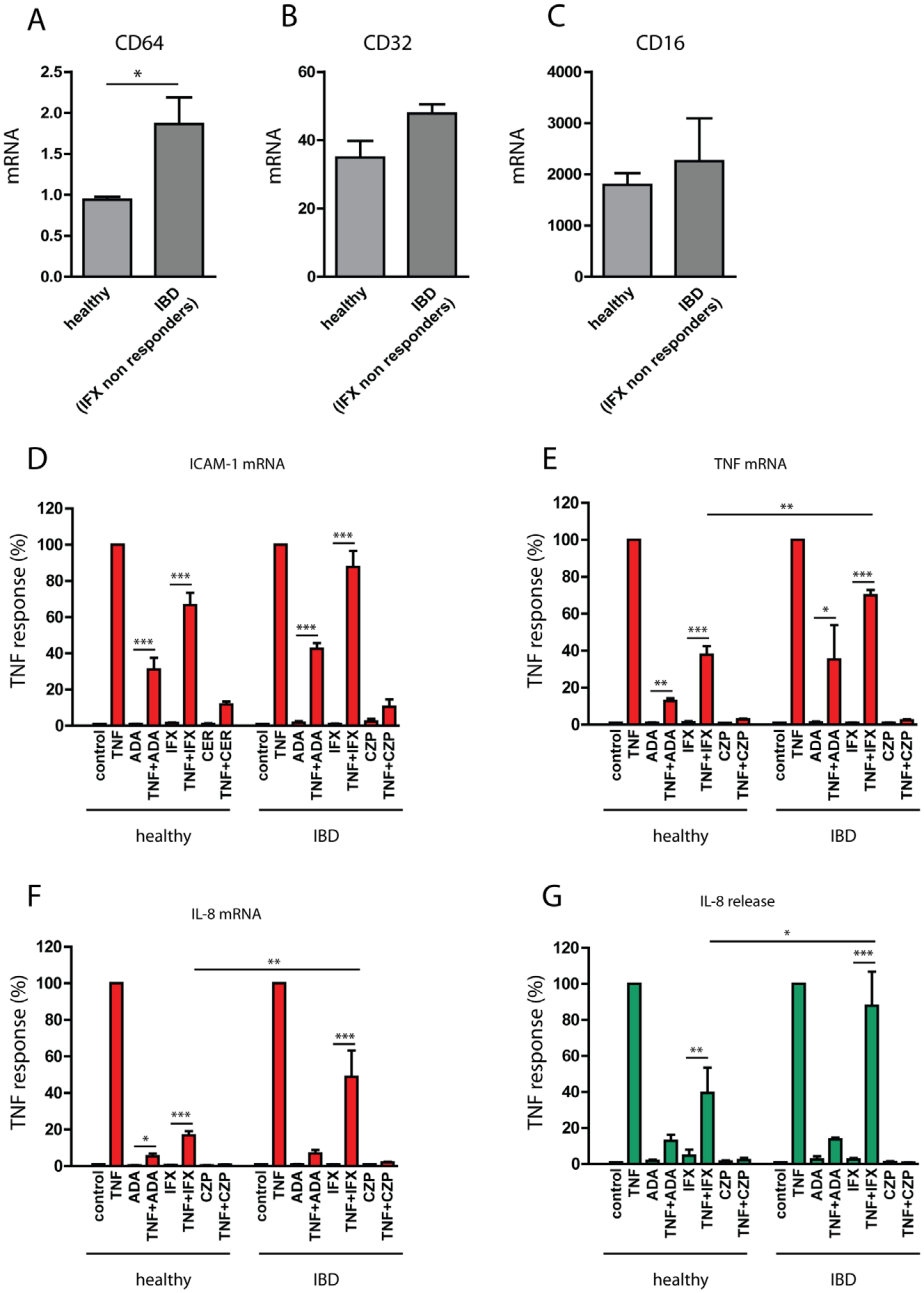
Adalimumab and infliximab, but not certulizumab-pegol have limited efficacy in PBMCs. Peripheral blood of healthy donors (n = 4) and IBD patients (n = 3) was treated with different anti-TNFs and subsequently with soluble TNF to induce pro-inflammatory responses in PBMCs and plasma. The mRNA expression levels of CD64 (A), CD32 (B) and CD16 (C) Fc gamma receptors in healthy individuals and IBD patients were assessed by RT-PCR as compared to THP-1 cells (set to 1). The extent of inhibition by each drug was determined by measuring mRNA levels of ICAM-1 (D), TNF (E), and IL-8 (F) after 2 h of TNF stimulation, as well as by the release of IL-8 after 24 h of combined TNF and anti-TNF stimulation (G). The inhibitory efficacies were determined after eliminating intra-individual differences by setting the TNF-induced responses to 100% for each donor and target (H–K). Error bars represent SEM. *p<0.05; **p<0.01; ***p<0.001.

To investigate the role of Fc receptors in modulating the inhibitory efficacy of anti-TNFs in the setting more closely resembling *in vivo* situation, we next tested these compounds *ex vivo* in peripheral blood obtained from healthy donors and IBD patients (Table S1). PBMCs express high levels of all Fc gamma receptors (Figure 2A–C), including low affinity Fc receptors CD16 and CD32, which are not expressed in THP-1 cells. We observed that PBMCs from IBD patients who initially did not respond to IFX therapy displayed significantly higher levels of CD64 and slightly elevated levels of both CD32 and CD16 as compared to healthy individuals, which was partially supportive to the observations reported earlier [22]. Interestingly, as compared healthy donors, both ADA and IFX had clearly limited efficacies in blocking TNF-mediated responses in blood of IBD patients as measured by elevated production of ICAM-1, TNF and IL-8 mRNAs (Figure 2D–F) as well as release of IL-8 in serum (Figure 2G). Interestingly, ADA, which was initially effective in THP-1 cells, induced pro-inflammatory responses in PBMCs when incubated with TNF. Strikingly, certolizumab-pegol, which lacks an Fc fragment, was completely effective, suggesting the involvement of Fc receptors/fragments in the observed induction of pro-inflammatory responses.

### IFX-TNF complexes activate Fc receptors and induce the expression of inflammatory targets via unique tyrosine phosphorylation signaling

**Figure 3:**
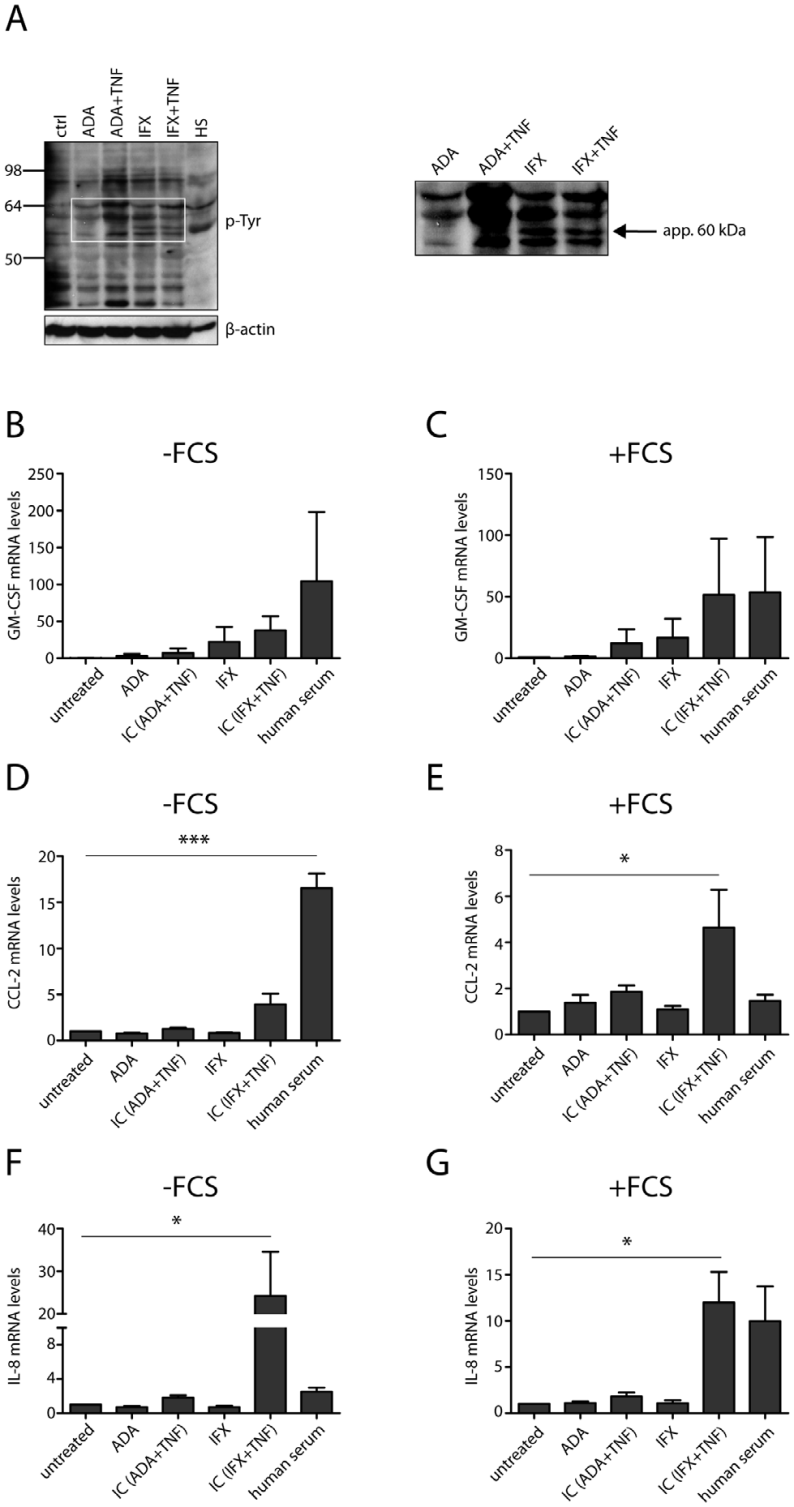
Infliximab is more potent in activating Fc receptor CD64 than adalimumab. Both ADA and IFX immune complexes were added to THP-1 cells to activate CD64 receptor in the presence (+FCS) or absence (−FCS) of serum. ADA/TNF complexes were more potent in inducing phospho-tyrosine signaling (A), but IFX either alone or in complex with TNF induced phosphorylation of distinct tyrosine residues (enlarged insert, right panel). Activation of CD64 downstream signaling leads to elevated transcription of GM-CSF (B, C), MCP-1 (D, E) and IL-8 (F, G) genes. Human serum (HS) was used as a positive control for activation of Fc gamma receptor(s). Error bars represent SEM. *p<0.05; **p<0.01; ***p<0.001.

In order to get more insight into possible molecular mechanism(s), which could contribute to the observed differences between ADA and IFX, we next investigated the activation of Fc receptors in a setting where anti-TNF IgGs and TNF were pre-incubated to form immune complexes prior addition to the cell cultures. Interestingly, we observed that ADA, either alone or in complex with TNF, was more potent in inducing phospho-tyrosine signals than IFX (Figure 3A), suggesting stronger Fc receptor binding and activation. Strikingly, IFX, either alone or in complex with TNF, was able to trigger phosphorylation of different tyrosine residues than ADA-TNF complexes as indicated by the appearance of additional bands (Figure 3A, enlarged insert). These signals were propagated to induce the transcription of target genes typically associated with responses triggered by IgGs, such as GM-CSF, MCP-1 and IL-8 (Figure 3B–G), which is consistent with the observations reported elsewhere [23]–[25]. Interestingly, binding of IFX-TNF complexes to CD64 on the surface of THP-1 cells triggered also the induction of IL-8, which explains at least partially, why IFX did not completely attenuate TNF-induced expression of this target gene (Figure 1D).

### Blocking Fc receptors with IgG1 molecules prevents activation of Fc receptors by infliximab and partially restores its efficacy

**Figure 4:**
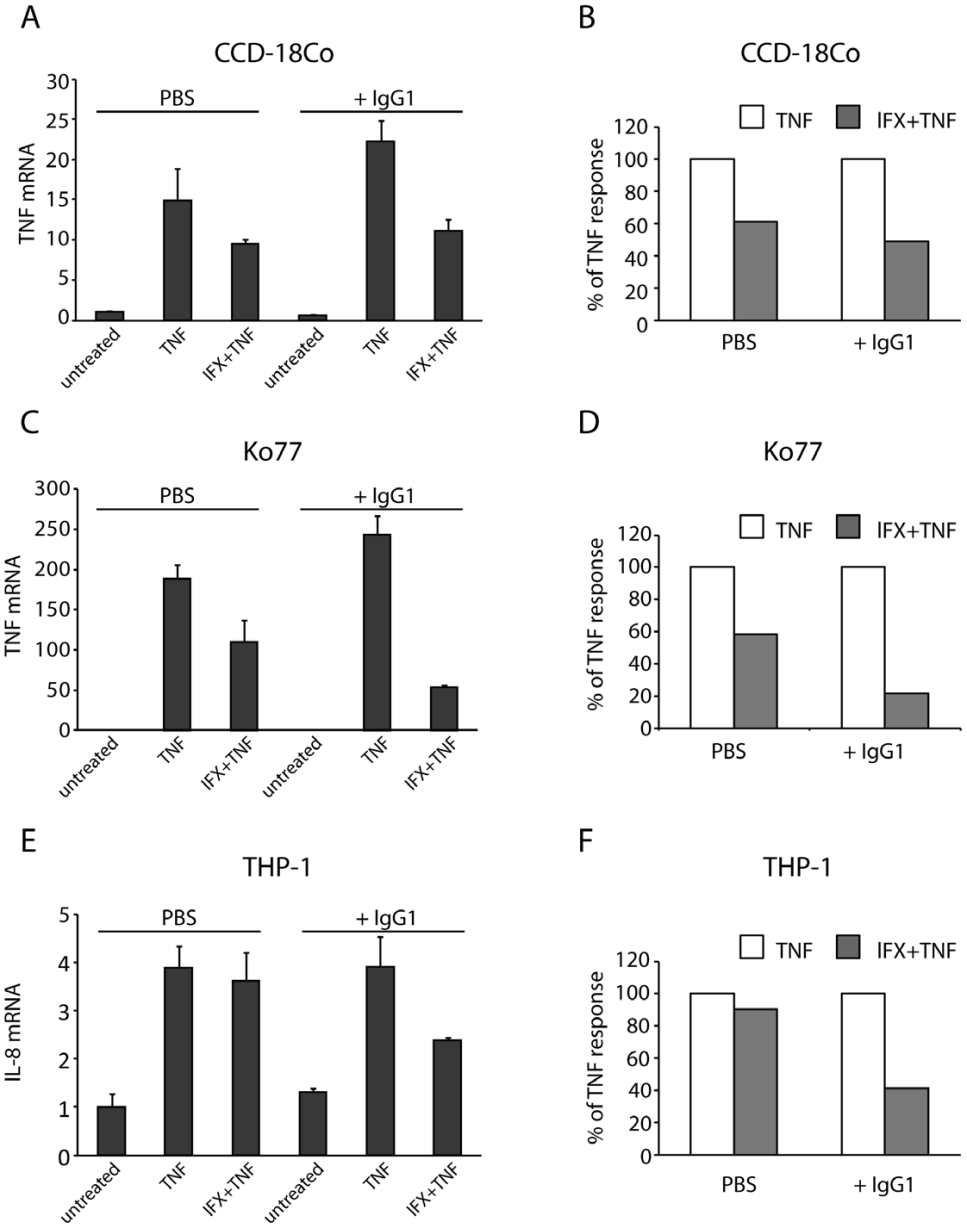
Blocking of Fc receptors with IgG1 partially restores the efficacy of infliximab. CCD-18Co fibroblasts (A), Ko77 fibroblasts (C) and THP-1 cells (E) were pre-incubated with human IgG1 isotype (10 µg/ml) prior to subsequent incubation with infliximab (1 µg/ml) and TNF (50 ng/ml). Columns show mean values from a single, representative experiment measured in triplicates. Error bars indicate SD of three measurements. The recovery of anti-TNF activity was also evaluated as percentage of inhibition of TNF-induced responses for each cell line (B, D, F).

In order to investigate whether binding to Fc receptors can indeed influence the inhibitory efficacy of infliximab, we pre-incubated the cells with human serum containing high amounts of IgGs prior to stimulation with anti-TNF therapeutics. As expected, human serum improved the efficacy of infliximab in case of both fibroblasts and THP-1 cells (Figure S6), but the extent of this effect was surprisingly low, which could possibly be explained by unwanted interactions with other serum components and/or the influence of other immunoglobulin (sub)classes present in serum. In order to avoid this, we performed a similar experiment exclusively in the presence of IgG1 isotype (Figure 4). Interestingly, although the recovery of efficacy was only 13% in case of CCD-18Co fibroblasts (Figure 4A and B), the effects of IgG1 in case of Ko77 fibroblasts and THP-1 cells were more pronounced (Figure 4C–F).

### Fc and Fab fragments of infliximab contribute differently to its inhibitory activity in intestinal fibroblasts and monocytic THP-1 cells

**Figure 5:**
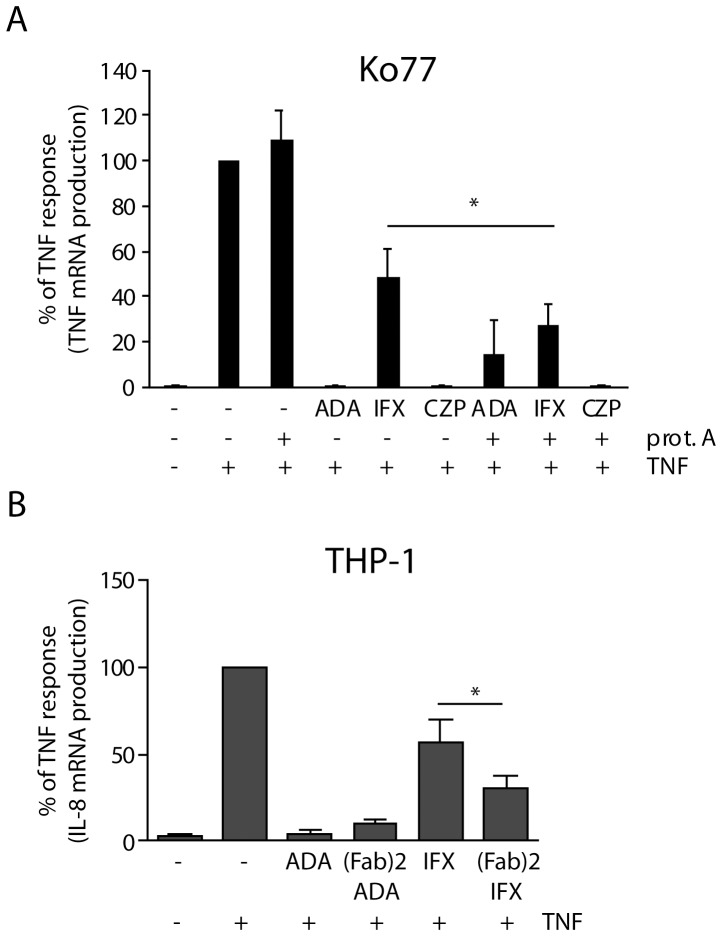
Fc and Fab fragments of infliximab contribute differently to its inhibitory efficacy. Blocking of Fc fragments of adalimumab and infliximab changes their inhibitory efficacy in Ko77 fibroblasts (A). Graph shows mean values from four independent experiments measured in triplicates. Error bars represent SD. Fab fragments of anti-TNFs have different inhibitory efficacies as compared to the whole IgG1 molecules in THP-1 cells (B). Columns represent mean values of three independent experiments measured in triplicates and set to 100 percent for TNF-treated cells. Error bars indicate SEM. *p<0.05.

In order to verify the role of Fc part of infliximab in limitation of its efficacy, we blocked the Fc fragments using protein A sepharose beads. To test the binding of anti-TNF antibodies to protein A sepharose beads, we employed standard SDS-PAGE/Coomassie approach (Figure S7,A). Once the optimal binding conditions were established, we blocked the Fc regions of both ADA and IFX by protein A sepharose beads and performed cell treatments using CD64-expressing CCD-18Co fibroblasts. Interestingly, blocking Fc fragments inflicted different effects on the inhibitory efficacy of both anti-TNFs IgGs: adalimumab partially lost its inhibitory potential, while infliximab's efficiency significantly improved (Figure 5A). In order to investigate the involvement of Fab fragments, we employed a protocol of proteolytic digestion where both Fab and Fc fragments were separated after 60 min of digestion with IdeS (Figure S7,B) and Fc fragments were removed from the reaction mixture using protein A sepharose beads (Figure S7,C). Interestingly, when isolated Fab fragments were used to block TNF-mediated responses in THP-1 cells, we found statistically significant recovery in case of infliximab and 6% loss-of-activity in case of adalimumab (Figure 5B). When the same experiment was performed using Ko77 fibroblasts, the observed changes had the same trends, but with minimal impacts (Figure S7,D).

### Changes in expression levels of CD64 influence the inhibitory efficacy of infliximab

**Figure 6:**
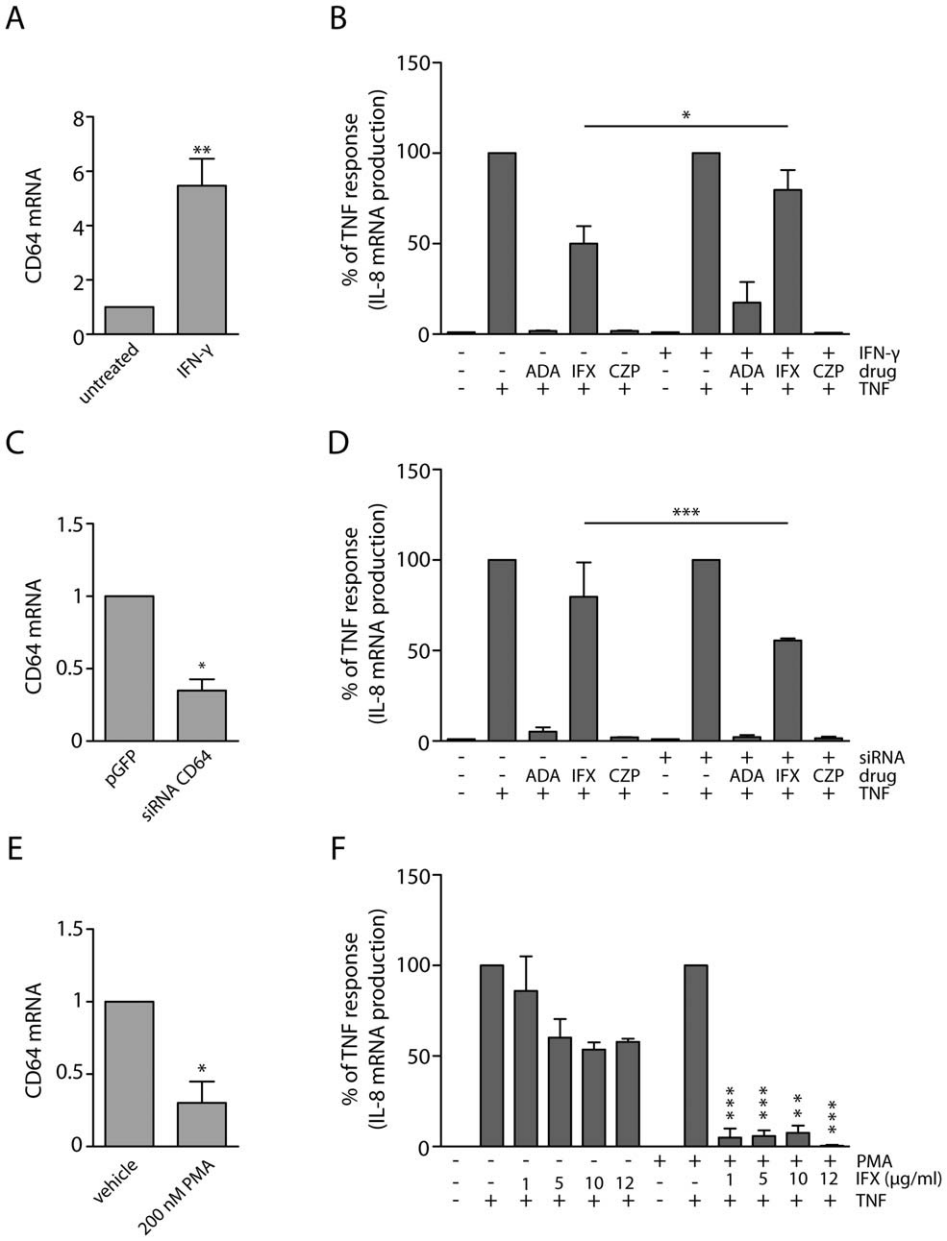
Changes in expression levels of CD64 affect the efficacy of infliximab in THP-1 cells. Treatment with IFN-γ elevates the mRNA expression levels of CD64 (A), which result in the loss of efficacy of IFX and ADA (B). Decreased expression levels of CD64 (C) correlate with increased inhibitory efficacy of IFX (D). PMA treatment decreases the expression levels of CD64 (E), which results in complete recovery of efficacy of IFX (F). Graphs show mean values of at least three independent experiments measured in triplicates and columns are set to 100 percent for TNF-treated cells. Error bars indicate SEM. *p<0.05.

In order to confirm that Fc fragments of ADA and IFX indeed modulate the efficacies of these drugs, we elevated the expression of high affinity IgG receptor CD64 prior to treatments. To this end, cells were pre-treated with IFN-γ for 48 h, followed by short-term incubation with anti-TNFs and treatment with TNF. Consistently with the existing data for both monocytes [26] and THP-1 cells [27], IFN-γ treatment resulted in elevation of CD64 expression on both mRNA and protein levels (Figure 6A and Figure S8, B). This was not the case for fibroblasts, probably due to the fact that the receptor already overexpressed upon CMV infection is less likely to further respond to cytokine treatment (Figure S8,A). Interestingly, elevated levels of CD64 correlated with a decrease in the efficacy of IFX in THP-1 cells (Figure 6B). In contrast to THP-1 cells, treatment of Ko77 fibroblasts with IFN-γ did not change the efficacy IFX (Figure S8,F), most likely due to the lack of its inducing effect on CD64 expression (Figure S8,A and S8,E). This could be due to previously reported suppressive effect of CMV on JAK/STAT pathway, which is crucial for propagating IFN-γ-induced responses [28]. This phenomenon was specific only for CD64, since the mRNA expression levels of other IFN-γ target genes, such as ICAM-1, were clearly induced upon treatment with this cytokine (Figure S2,F and S8,C). To confirm whether CD64 is directly involved in mediating inhibitory efficacy of IFX, we next suppressed the expression of this receptor in THP-1 cells, before incubation with anti-TNFs. Specific siRNAs were able to decrease the expression of CD64 by 70% at the mRNA level (Figure 6C) and by 60% on protein level (Figure S9,A). Although treatment with siRNA improved the inhibitory efficacy of infliximab (Figure 6D), the extent of this change was only moderate, possibly due to limited effect of siRNA on the protein level. Another possibility would be the lack of suppressing effect on the other CD64 isoforms, which could be expressed in THP-1 cells, or because some other Fc receptors, e.g. other isoforms of CD64 and/or CD16, may interact with infliximab in our experimental set-up. To overcome these limitations, we used phorbol-12-myristate-13-acetate (PMA), a potent inducer of differentiation and a known modulator of the CD64 expression in THP-1 cells [29]. As expected, we observed a significant decrease in CD64 mRNA levels upon PMA-induced differentiation (Figure 6E and Figure S9,B), which correlated with an increase of macrophage-specific marker CD68 and another Fc gamma receptor CD16 (Figure S9,C and S9,D). Consistently with our working hypothesis, PMA-induced decrease of CD64 expression correlated with a complete recovery of the inhibitory efficacy of infliximab, even at lower concentrations of the drug (Figure 6F).

### High mRNA expression levels of Fc receptors in inflamed colonic tissue correlate with the loss of response to infliximab in IBD patients

**Figure 7:**
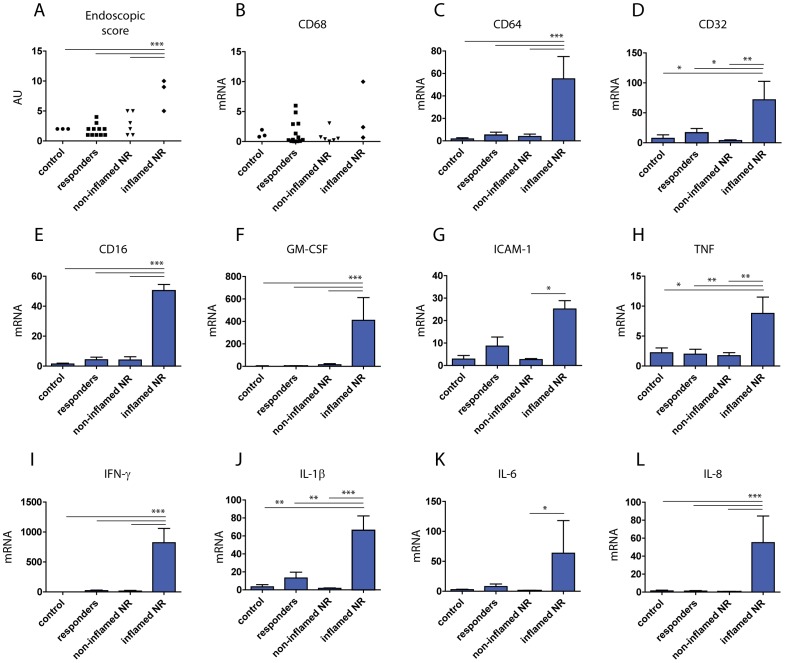
Loss of response to infliximab correlates with increased levels of CD64 in IBD intestines. The level of intestinal inflammation was assessed independently by endoscopic score and mRNA expression levels of pro-inflammatory target genes. The levels of Fc receptors were assessed in colonic biopsies from IFX responders (n = 13) tissues and compared to IFX non-responders in both non-inflamed (n = 6) and inflamed (n = 3) tissues. IL-18 mRNA was used as a specific negative control, since it is not elevated upon stimulation with pro-inflammatory cytokines (not shown). The expression levels of each gene were normalized to those of a housekeeping gene β-actin. *p<0.05; **p<0.01; ***p<0.001.

To investigate the role of Fc receptors in the responsiveness towards anti-TNF therapy we screened colonic biopsies of IFX responders and non-responders together with the healthy control subjects for the mRNA expression levels of various inflammatory targets and Fc receptors (Figure 7). Endoscopic score was assessed and presented in Figure 7A. The mRNA levels of all pro-inflammatory targets were significantly higher in inflamed tissues of IFX non-responders as compared to IFX responders, which was consistent with the lack of the therapeutic response outcome assessed by colonoscopy examination (Table S2 and Figure 7A). Interestingly, the colonic mRNA levels of CD68 were not significantly different between IFX responders and IFX non-responders (Figure 7B), suggesting that increased infiltration of the immune cells, such as macrophages, was not responsible for the loss of response to IFX, which was suggested recently [16]. Strikingly, inflamed colonic biopsies from IFX non-responders displayed significantly higher mRNA levels of all Fc gamma receptors as compared to the non-inflamed tissues of IFX responders (Figure 7C–E), along with relevant inflammatory mediators (Figure 7F–L).

## Discussion

In the present study we propose a new mechanism that modulates the inhibitory efficacy of infliximab, which is dependent on both its Fc fragment and the presence of high affinity Fc gamma receptor CD64. We have demonstrated that IFX was inefficient in cell types expressing CD64 including fibroblasts, monocytic THP-1 cells and different subsets of immune cells (PBMCs). We demonstrated that IFX was more potent in activating CD64 by inducing the transcription of pro-inflammatory genes, such as IL-8 and MCP-1. This was in striking contrast to CZP, which blocks TNF, but lacks Fc region. Fab fragments of IFX have statistically higher inhibitory efficacy, compared to the whole IgG molecules and both blocking Fc parts of this drug and changes in expression of CD64 correlate with the changes in its ability to block TNF-induced responses. Perhaps most importantly, we have shown that IFX non-responders express higher mRNA levels of CD64 in their inflamed colonic tissues.

**Figure 8:**
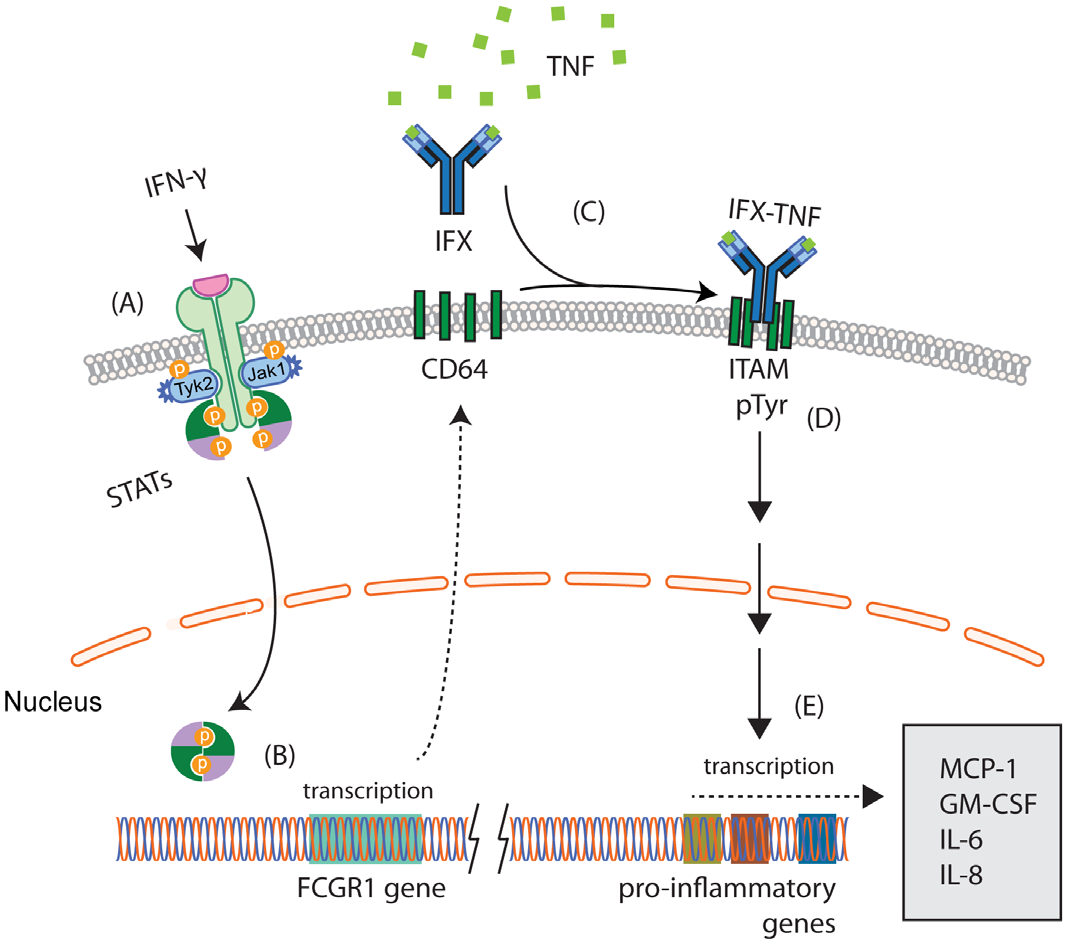
Schematic representation of the working model. IFN-γ induces its receptor downstream signaling involving JAK and STAT proteins (A). STAT dimers elevate the transcription of the *CD64 (FCGR1)* gene (B). Translated CD64 protein is targeted into plasma membrane, where it resides in an inactive state until binding immune complexes IFX-TNF (C). Propagation of downstream signaling (D) occurs through immunoreceptor tyrosine-based activation motif (ITAM) leading to the transcription of relevant target genes (E) via parallel activation of p38, JNK and ERK kinases [58]. Production and release of IL-8 and other pro-inflammatory factors contribute to the limited outcome of IFX treatment.

In agreement with our findings, it has been shown that ADA and IFX bind to Fc receptor-expressing THP-1 cells in a manner that is dependent on the addition of the human recombinant Fc fragment [14]. At this point it cannot be excluded that binding of IFX to Fc receptor(s) alone limits the ability of this drug to block soluble TNF. Nevertheless, it is more likely that IFX-TNF complexes, which form during neutralization of TNF, are responsible for the limited anti-inflammatory effect via direct activation of Fc receptor downstream signaling (Figure 8). Adalimumab appears to have different mechanism in activating Fc receptors as compared to infliximab and this seems to play role in its overall anti-inflammatory outcome in THP-1 cells, but not PBMCs (Figure 3 and Figure 2, respectively).

Our results imply that there are differences between tested anti-TNF IgGs in their Fc fragments, which could be directly responsible for the observed phenomena. Different glycosylation patterns within Fc fragment might be responsible for the observed differences in the efficacies between ADA and GOL (Figure 1 and Figure S5). It is known that IFX contains more sialyl and core fucose residues than ADA [30]. It can be anticipated that these differences have direct consequences on the extent of the Fc receptor(s) activation, which are expressed on the surface of the implicated cell types, such as CMV-positive fibroblasts and monocytes/macrophages (Figure 2). These and/or other glycal residues could potentially translate to the clinical efficacy as suggested elsewhere [31]. Removal of N-linked glycal residues did not show any influence on the efficacy of ADA and IFX in our experimental setup (Figure S10), suggesting that activation of CD64 in our experimental setting is independent on the presence of glycal residues linked to Fc region. In line with this notion, binding to Fc receptors could lead to formation of large immune complexes specific for different anti-TNFs and when bound to the surface of cells expressing Fc receptor(s), it could in turn reduce the diffusion of free anti-TNF IgG molecules and thereby limit the amount of anti-TNF molecules capable of binding soluble TNF and/or anti-TNFs engaged in ADCC. Either way, it can be excluded that mTNF could be responsible for the observed limitations of IFX, since we did not detect it in any of cell lines used in the study (Figure S4, D). Despite this attractive hypothesis, until now there are no studies supporting such mechanism and our investigation is the first one, which, indicates that, this could be the case.

Recently, it has been shown that certolizumab-pegol does not show significant therapeutic efficacy as compared to placebo in a large cohort of IBD patients indicating the importance of Fc fragment in providing desired therapeutic effect [32]. Despite the fact that we observe clear Fc-dependent negative effect of IFX in our experimental system, at this point one cannot draw any definite conclusions about which of the anti-TNFs would have overall best therapeutic efficacy, since it greatly depends on many factors, such as individual differences in biodistribution, state of the disease and cell-drug interactions, which vary among IBD patients. On the other hand, CZP induces a remission of the disease in patients with elevated CRP levels. It is therefore impossible to estimate if Fc fragment has a non-redundant role in providing the therapeutic effect in all IBD patients.

Recently, CD64 has been implicated as a modulator of responses against microbial challenge in the context of mucosal inflammation in the intestine [33]. Additionally, CD64 and toll-like receptor 4 have been reported to be up-regulated in CD patients [34] and that this correlates with the clinical and biological parameters of inflammation in patients with IBD [35]. These observations point out for the role of Fc receptors as diagnostic markers and as potential modulating factors for immunoglobulin-based drugs, similarly to what has been suggested for abatacept and CD64 [36]. In line with these ideas, it has been demonstrated that there is a correlation between the responsiveness towards infliximab and polymorphisms in the FcRIII receptor isoforms [37], [38]. Similar association was found in the case of rheumatoid arthritis [39], [40], which could support the current hypothesis that Fc receptors can influence the outcome of IFX therapy.

Interestingly, we observed a correlation between IFN-γ-induced CD64 expression and the loss of inhibitory efficacy of IFX *in vitro* (Figure 6A and 6B). These data were strongly supported by the fact that in contrast to IFX responders, inflamed tissues of IFX non-responders express very high levels of IFN-γ, which elevates the expression levels of CD64 [26], [27]. Strikingly, among all tested target genes the most highly elevated cytokines in IFX non-responders' inflamed colonic tissues are the ones which are generated upon binding to activating Fc gamma receptors (CD64 and CD16), namely GM-CSF, IL-6 and IL-8 (Figure 7). It is still not clear whether increased expression levels of Fc gamma receptors detected in IFX non-responders are a cause or a consequence of the lack of therapeutic effect of this drug. Activation of Fc gamma receptors alone triggers the production of MCP-1, IL-6 [41], and GM-CSF, which in turn leads to generation of autoreactive T-cell responses [42]. As CD64 and other Fc receptors are expressed by many immune cells, including monocytes [43], most of the antigen-presenting cells and phagocytic cells [44], the efficacy limitations may be relevant at different sites of inflammation, such as blood, arthritic joints and gastrointestinal tract. In support of this notion, it has been reported that another Fc receptor, CD16, is elevated in IBD monocytes [45], [46], macrophages [47], and natural killer cells [48] residing in the inflamed intestine. In line with our findings it may indicate that monocytes and/or related cell types, such as dendritic cells and macrophages, could be potential interacting partners for IFX complexes and/or possibly other anti-TNF IgGs at sites of inflammation. Elevated soluble forms of this receptor have been detected in gut lavage fluid of IBD patients [49], which in turn suggests that interaction between anti-TNF IgGs should attract more attention with regard to possible consequences for the therapy outcome.

In this study, we have also confirmed that intestinal fibroblasts infected with CMV express relatively high amounts of high and low affinity IgG receptors suggesting a correlation between CMV infection and responsiveness towards IFX. Depending on the subtype and the severity of the disease the occurrence of CMV infection in the intestine of IBD patients varies between 6 and 64%, when determined using immunohistochemistry as a ‘golden standard’ method of detection [50]. However at the same time other studies indicate that the prevalence can vary greatly among cohorts and could crucially depend on the detection method [51], [52]. CMV-induced FcR has strong preference to IgG1 as compared to other IgG sub-classes [53]. In the context of these findings, it has been recently demonstrated that the presence of CMV is positively correlated with the loss of response to IFX in IBD patients [54]. This observation was very supportive to our data emphasizing the role of CD64 in the clinical efficacy of IFX and possibly other anti-TNFs. Future studies will be needed to evaluate the role of CD64 and potentially other Fc receptors in modulating efficacy of anti-TNF drugs in more controlled clinical setting thereby creating a perspective for personalized biological therapy for IBD patients.

## Materials and Methods

### Ethics statement

Human intestinal biopsies and blood samples were collected at the Clinic of Gastroenterology and Hepatology USZ in accordance with the guidelines of ethical committee of Canton Zurich. The committee specifically approved this study. Written consents were taken from all the patients.

### Patients' samples

We analyzed biopsy specimens from 22 IBD patients (age 23–57 years), including 14 CD and 8 UC patients. Colonic biopsies were taken from the sigmoid colon in all patients except one CD patient in whom the sigmoid had been removed earlier after perforation. Specimens were taken from either inflamed or non-inflamed regions. Most IBD patients were immunosuppressed at the time of colonoscopy (See Table S2). As control we analyzed biopsies from sigmoid colon of healthy donors (age between 66 and 77), who underwent surveillance colonoscopy after earlier polypectomy. Detail description of patients' characteristics is listed in Table S2.

### Antibodies, Taqman assays, oligonucleotide primers and other reagents

Human recombinant IFN-γ (I3265), mouse monoclonal anti-human β-actin antibody (A5441), DAPI (D9564) and PMA (P1585) were purchased from Sigma-Aldrich (Buchs, Switzerland). Recombinant TNF (C-63720) was from PromoCell/PromoKine (Heidelberg, Germany). Human recombinant IL-1β (407615) was purchased at Calbiochem-Merck Chemicals (Germany). Adalimumab was from Abbott (Baar, Switzerland), infliximab from Essex Chemie AG (Luzern, Switzerland), certolizumab-pegol from UCB Pharma (Bulle, Switzerland), and golimumab from Centocor Ortho Biotech Inc. (Horsham, PA). Mouse monoclonal anti-α-smooth muscle actin (SMA) antibody (ab40865) was from Abcam (Cambridge, UK), anti-villin antibody (MAB1639) from Chemicon (USA), and anti-CD16 (sc-70548), anti-CD32 (sc-53663), anti-CD64 (sc-1184), anti-TNFR1 (sc-7895) and anti-fibronectin (sc-9068) antibodies were from Santa Cruz Biotechnology (Santa Cruz, CA, USA). Anti-CD64 antibody (3860-1) used for detection of glycosylated band was obtained from Epitomics (Burlingame, CA). Human IgG1 isotype control (ALX-804-133) was purchased at Enzo Life Sciences (Plymouth Meeting, PA, USA). CCL2 (Hs00234240_m1), CSF2 (Hs00929873_m1), IL-1β (Hs00174097_m1), IL-6 (Hs00174131_m1), IL-8 (Hs00174103_m1), ICAM-1 (Hs00164932_m1), CD68 (Hs00154355_m1), FCGR1A (Hs00174081_m1), FCGR2A (Hs01017702_g1), FCGR3 (Hs00275547_m1), TNF (Hs00174128_m1) and β-actin (4310881E) human gene expression assays were provided by Applied Biosystems (Foster City, CA). For CMV detection we used primers specific for viral DNA polymerase UL54 [55], which were purchased from Microsynth (Balgach, Switzerland).

### Cell lines

Caco2*_BBE_*, CCD-18Co, and THP-1 cell lines were cultivated at 37°C in a 5% CO_2_ incubator. Both Caco2*_BBE_* and CCD-18Co cell lines were cultivated in high-glucose DMEM medium (Sigma-Aldrich) containing L-glutamine, sodium bicarbonate, 10% FCS, penicillin (100 U/ml) and streptomycin (100 µg/ml). THP-1 cells were cultured in RPMI-1640 medium (Sigma-Aldrich) supplemented with 10% FCS, penicillin (100 U/ml) and streptomycin (100 µg/ml). All cell lines were purchased from ATCC-LGC Promochem (Molsheim Cedex, France). Ko77, F141, F148, and F188 fibroblasts were isolated and cultivated as described elsewhere [56].

### Cytokine treatments and incubations with anti-TNF therapeutic antibodies

Cell lines were incubated with pro-inflammatory cytokines TNF, IL-1β, and IFN-γ at the concentration of 50 ng/ml. Concentrations of the anti-TNF IgG1s used in the experiments were 10 µg/ml (adalimumab, infliximab and golimumab) and 24 µg/ml for certolizumab-pegol. For inhibition experiments cells were pre-incubated with anti-TNF therapeutics for 60 min at 37°C prior to treatment with TNF (50 ng/ml) for another 60 min.

### RNA isolation, cDNA synthesis, and real-time PCR

RNA was isolated with QIAcube (QIAGEN, Hombrechtikon, Switzerland) using the protocol for animal cell isolation with DNAse digest, or alternatively using TRIzol reagent (Invitrogen, Zug, Switzerland). The amount and quality of RNAs were determined using ND-1000 spectrophotometer (NanoDrop Technologies, Wilmington, DE). Complementary DNAs were produced using High Capacity cDNA Reverse Transcription Kit (4368814, Applied Biosystems). Real-time PCR reactions containing 10 µl of Fast Universal Master Mix (Applied Biosystems) and 1 µl of a TaqMan gene expression assay (Applied Biosystems) in 20 µl of total volume. The cycle conditions were: stage 1 (50°C, 2 min); stage 2 (95°C, 10 min); stage 3: 40 repeats (95°C, 15 s; 60°C, 1 min). The expression levels of the target genes were normalized to the expression levels of β-actin using the comparative threshold cycle method.

### Protein concentration determination

Protein concentrations were determined using the BCA Protein Assay Reagent (Pierce, Wohlen, Switzerland).

### SDS electrophoresis and Western blotting

Protein electrophoresis was performed according to the standard protocol. Briefly, proteins were separated in 10% polyacrylamide gels (Tris/glycine) and transferred onto nitrocellulose membranes. For Western blotting, membranes were blocked with 5% solution of non-fat dried milk buffer in TBS-T (TBS+0.1%Tween) for 1 h at RT with gentle shaking. Primary antibodies were diluted in TBS-T containing 5% BSA and incubated with the membranes overnight at 4C°. Corresponding secondary antibodies were diluted in TBS-T buffer containing 5% blocking milk for 1 h at RT. Bands were detected by using ECL detection kit (GE Healthcare, Glattbrugg, Switzerland) and autoradiography. Densitometry analysis of the protein bands was performed using OptiQuant software.

### Immunofluorescence microscopy

CCD-18Co cells were plated onto sterile glass coverslips and cultured until full confluence. Prior to their fixation cells were washed once with PBS and fixed with 3.7% solution of formaldehyde for 30 min at RT. After washing three times with PBS, cells were permeabilized with 0.25% Triton X-100 for 15 min at RT prior to incubation with blocking solution (1% of bovine serum albumin (BSA) in PBS) for 1 h at RT, and subsequently with anti-fibronectin and anti-CD64 antibodies overnight at 4°C. Coverslips were incubated with corresponding secondary anti-rabbit Cy2 and anti-mouse Cy3 antibodies diluted in blocking solution. Samples were mounted using anti-fade mounting medium (DAKO, Glostrup, Denmark) and analyzed by fluorescence microscopy (Zeiss, Oberkochen, Germany).

### Incubation of peripheral blood with anti-TNFs and isolation of PBMCs

Fresh human peripheral blood (40 ml) was divided into eight parts for treatment with anti-TNFs and soluble TNF according to the protocol used for cell lines (see above). PBMCs were isolated in according to standard Ficoll (GE Healthcare) protocol. Briefly, diluted blood was overlayed onto Ficoll and centrifuged at 1700 rpm for 40 min. PBMCs from interphase were collected, washed once with PBS+0.1% BSA and harvested for RNA extraction. Detail description of blood samples from both healthy donors and IBD patients used for this set-up is listed in Table S1.

### Formation of anti-TNF/TNF immune complexes

Complexes of ADA or IFX and TNF were formed by incubation at 37°C for 30 min in PBS prior to addition to cells. The ratio of anti-TNFs (100 µg/ml) vs soluble TNF (500 ng/ml) was the same as for stimulation experiments (see above).

### Blocking of Fc fragments of therapeutic antibodies

Adalimumab and infliximab were incubated with protein A sepharose beads (GE Healthcare) for 30 min at RT prior to incubation with fibroblasts for 30 min at 37°C. Afterwards, the cells were treated with TNF for 1 h, harvested and processed for RNA isolation.

### Proteolytic digestion and deglycosylation of therapeutic antibodies

Adalimumab and infliximab were digested by IdeS endopeptidase as described elsewhere [57]. Anti-TNFs were incubated with IdeS (FabRICATOR®, Synovis, Sweden) according to manufacturer's protocol and processed for protein chip analysis (Agilent Technologies, USA). Purified Fab′ fragments were obtained by removing Fc fragments from the reaction mixture using protein A sepharose beads according to the manufacturer's protocol (General Healthcare, UK). Deglycosylation of anti-TNFs was performed using recombinant glycosidase (IgZERO®, Synovis, Sweden) according to manufacturer's protocol.

### SiRNA transfections

Transient downregulation of CD64 expression in THP-1 cells was achieved using Nucleofector® kit V (Amaxa, USA). Specifically, 10^6^ cells per condition were suspended in 100 µl of Nucleofector® solution and 6 µl of 50 µM siRNA mix containing three different siRNA sequences: s5069, s5068, and s230564 (Applied Biosystems/Ambion, USA) were added for 10 min at RT. Next, the samples were subjected to nucleofection using high viability protocol (V001) and grown in standard medium for 3 days.

### Statistical analysis

Statistical significance was determined by performing either ANOVA analysis with Tukey's multicomparison post-test or two-tailed paired t-test using Graph Pad Prism 5 software (GraphPad Software, La Jolla, CA).

## Supporting Information

Figure S1 **Ko77 and CCD-18Co fibroblast are CMV-positive.** (A) Real-time PCR amplification curves show the expression of viral UL54 mRNA in both fibroblastic cell lines used in the study. The specificity of the PCR products was confirmed by the kinetics of dissociation curves for β-actin (B) and UL54 (C).
(TIF)

Figure S2 **Intestinal epithelial cells, intestinal myofibroblasts and monocyte-macrophage cells can be efficiently treated with inflammatory cytokines to induce inflammatory responses.** Caco2*_BBE_* (A–C), CCD-18Co (D–F) and THP-1 (G–I) cell lines were treated with three different cytokines to induce pro-inflammatory responses. Cells were harvested at different time points to monitor the kinetics of mRNA production of different genes. Values on Y-axis represent mRNA expression levels relative to ß-actin. Columns represent the mean values of three measurements within a single, representative experiment. Error bars represent SD.
(TIF)

Figure S3 **Pro-inflammatory cytokines trigger specific signaling pathways in the cell lines used in the study.** (A) IFN-γ, but neither TNF nor IL-1β, induces phosphorylation of STAT1 in intestinal epithelial Caco2*_BBE_* cells. (B) TNF induces phosphorylation of CREB, ATF-1 and p38 MAPK proteins in intestine-derived fibroblasts CCD-18Co. (C) TNF, but not IFN-γ, activates NF-κB in Caco2*_BBE_* cells as measured by electrophoretic mobility shift assay. Cells were treated with two different pro-inflammatory cytokines to test the specificity of the binding to the NF-κB-specific radiolabelled probe. Maximum activation was observed after 60 min. Addition of anti-p65 antibodies shifts the size of the protein-DNA complexes towards higher molecular weight, showing the specificity of the protein binding to the probe. (D) IL-1β activates NF-κB in Caco2*_BBE_* cells as measured by EMSA. Maximum activation was observed after 30 min. All cytokines were used at the concentration of 50 ng/ml.
(TIF)

Figure S4 **Infliximab has limited efficacy in fibroblasts isolated from CD patients.** (A) Fibroblasts isolated from CD patient (MC153) and (B) isolated from fistulizing CD patient (F188) were incubated with either adalimumab or infliximab before treatment with TNF. Columns represent the mean values of three measurements within a single, representative experiment relative to ß-actin. Error bars represent SD. Caco2*_BBE_* cells, intestinal fibroblasts and THP-1 cells express Fc receptors (C), but not mTNF (D). Recombinant TNF was used as a positive control (17 kDa). M: Molecular weight marker.
(TIF)

Figure S5 **Golimumab displays reduced inhibitory efficacies in intestinal fibroblasts and THP-1 cells, but not in intestinal epithelial Caco2** ***_BBE_*** **cell line.** (A) THP-1 cells (B) Caco2*_BBE_*, (C) Ko77, and (D) CCD-18Co cells were pre-incubated with golimumab and subsequently treated with TNF. The graphs show the results of a single experiment, measured in triplicates. Error bars represent SD.
(TIF)

Figure S6 **Human serum changes the efficacy of IFX on fibroblasts and monocytes.** (A) CCD-18Co fibroblasts were pre-incubated with human serum for 30 min prior to treatment with anti-TNF therapeutics and TNF. Graph shows result of a single experiment measured in triplicate. Error bars indicate SD of three measurements. (B) Quantification of TNF inhibition by infliximab in CCD-18Co fibroblasts expressed as percentage. (C) THP-1 cells were pre-incubated with human serum for 30 min prior to treatment with anti-TNF therapeutics and TNF. Graph shows result of single experiment measured in triplicates. Error bars indicate SD of three measurements. (D) Quantification of TNF-induced response inhibition by infliximab in THP-1 cells expressed as percentage. Graphs show the results of single experiment measured in triplicates.
(TIF)

Figure S7 **Blocking of Fc and isolation of Fab fragments can be successfully applied for testing the efficacies of anti-TNFs.** (A) Both adalimumab and infliximab, but not certolizumab-pegol bind to protein A sepharose beads. (B) Fab and Fc fragments can be produced by incubation with recombinant immunoglobulin-degrading enzyme of Streptococcus (IdeS, FabRICATOR®). (C) After proteolytic digestion Fab fragments of ADA and IFX were removed from the reaction mixture by immunoprecipitation using protein A sepharose beads. (D) Ko77 fibroblasts were pre-treated with either intact IgG1 molecules or Fc-purified digestion mixture containing Fab′ fragments of ADA or IFX and subsequently stimulated with TNF. The percentage of TNF inhibition was assessed based on the production of TNF mRNA by RT-PCR.
(TIF)

Figure S8 **IFN-γ does not induce CD64 mRNA expression and does not influence inhibitory efficacy of IFX in Ko77 fibroblasts.** mRNA expression levels of CD64 in Ko77 fibroblasts (A) and THP-1 cells (B) upon IFN-γ treatment. IFN-γ-induced progressive elevation of ICAM-1 mRNA levels in Ko77 (C), which was similar to the effect observed for CCD-18Co fibroblasts (Figure S2,F). (D) mRNA levels of CD64 in Ko77 cells after 48 hours of IFN-γ treatment. (E) Protein levels of CD64 in Ko77 and THP-1 cells after 48 hours of IFN-γ treatment. (F) Pre-incubation with IFN-γ had no impact on inhibitory efficacy of IFX in Ko77 fibroblasts. The graph shows results from a single experiment measured in triplicates. Error bars indicate SD of triplicates.
(TIF)

Figure S9 **The expression levels of CD64 can be decreased by either siRNA or PMA treatment in THP-1 cells.** (A) Western blot analysis of CD64 in pGFP- and siRNA-transfected cells. The mRNA expression levels of CD64 (B), macrophage-specific marker CD68 (C) and CD16 (D) analyzed 48 h after the treatment with different amounts of PMA. The graphs show results from a single representative experiment measured in triplicates. Error bars indicate SD of triplicates.
(TIF)

Figure S10 **Glycosylation at Asn297 does not influence the inhibitory efficacy of anti-TNF therapeutic antibodies.** (A) Both adalimumab and infliximab were deglycosylated at Asn297 as indicated by the shift in molecular mass in SDS-PAGE and Coomassie staining. This modification did not result in significant change in the inhibitory efficacy of anti-TNF antibodies in either Ko77 fibroblasts (B) or in monocytic THP-1 cells (C). The graphs show results from a single experiment measured in triplicate. Error bars indicate SD of triplicates. ADA(d): de-glycosylated adalimumab; IFX(d): de-glycosylated infliximab.
(TIF)

Table S1 **Basic characteristics of peripheral blood donors used in the study.** 5-ASA: 5-aminosalicylic acid, IFX: infliximab.
(TIF)

Table S2 **Basic description of human intestinal specimens used in the study.** 5-ASA: 5-aminosalicylic acid; IFX: infliximab; ADA: adalimumab; ABA: abatacept; VED: vedolizumab; MTX: methotrexate; CZP: certolizumab-pegol; nd: not determined; N/A: not applicable.
(TIF)
