# Supplementary figures and images for: Fc Gamma Receptor CD64 Modulates the Inhibitory Activity of Infliximab
Source: PLoS One. 2012 Aug 24;7(8):e43361. doi: 10.1371/journal.pone.0043361 (PMC3427356; doi:10.1371/journal.pone.0043361)

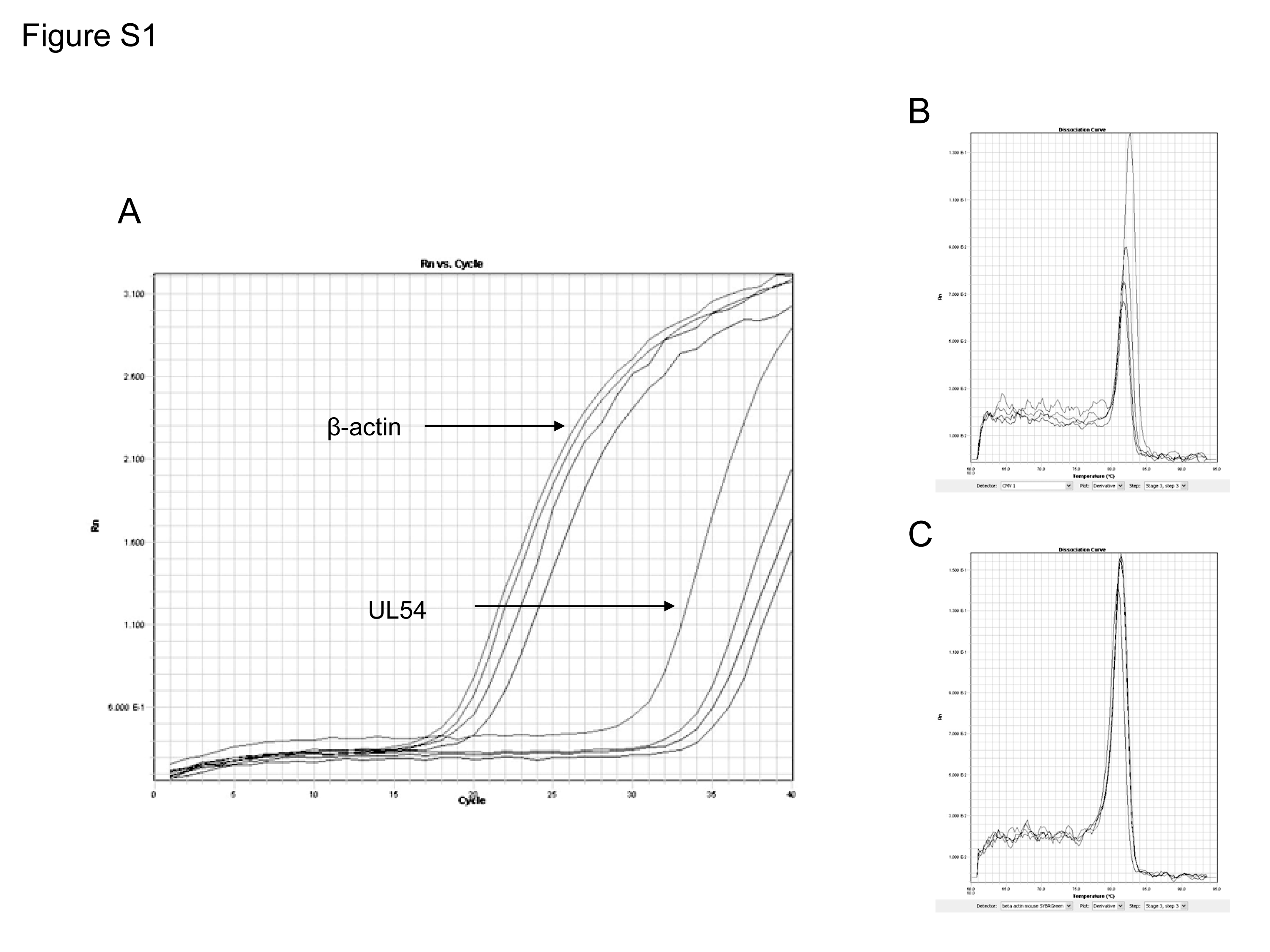

Supplement: Figure S1 — Ko77 and CCD-18Co fibroblast are CMV-positive. (A) Real-time PCR amplification curves show the expression of viral UL54 mRNA in both fibroblastic cell lines used in the study. The specificity of the PCR products was confirmed by the kinetics of dissociation curves for β-actin (B) and UL54 (C). (TIF) [file pone.0043361.s001.tif]

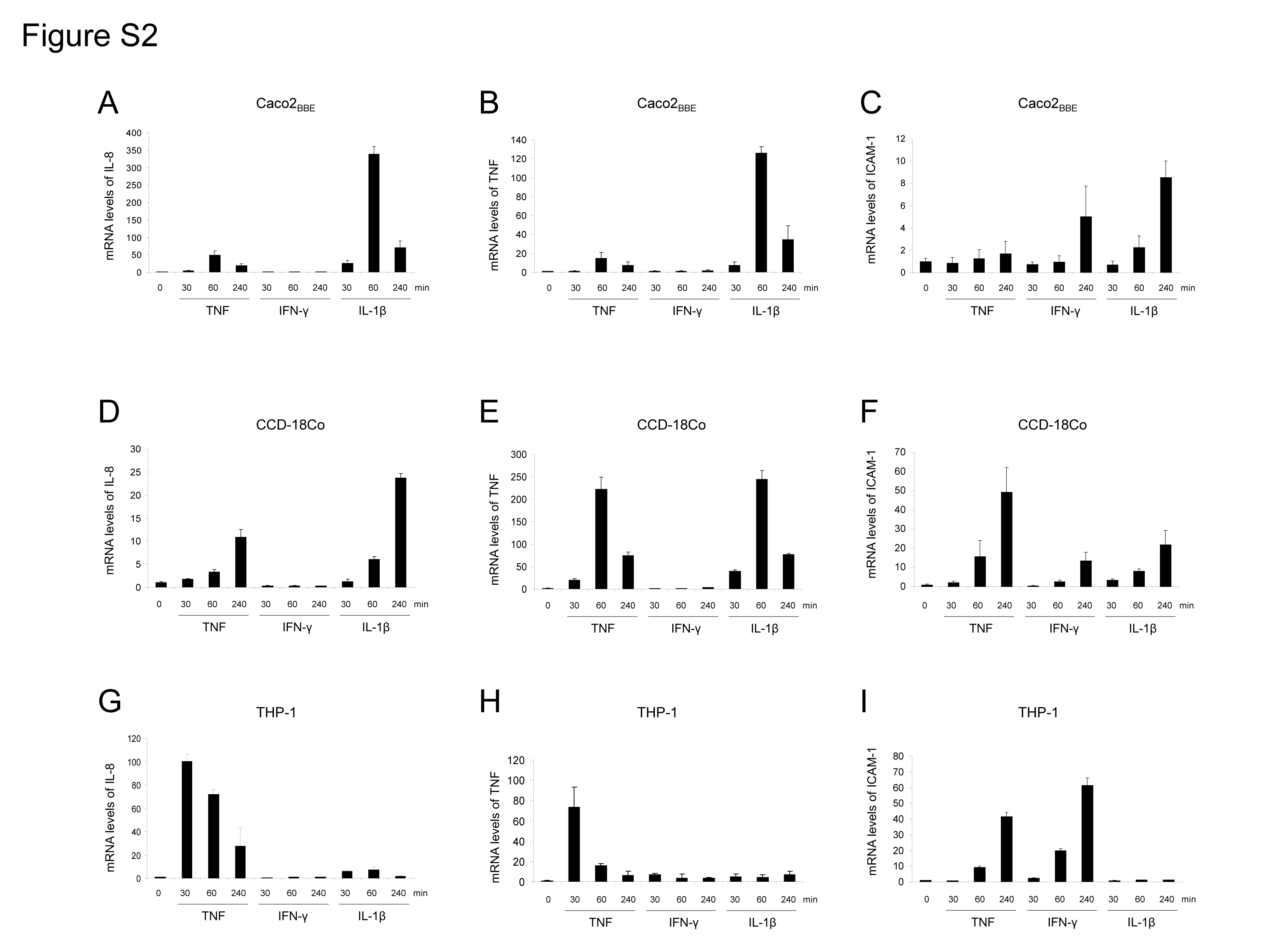

Supplement: Figure S2 — Intestinal epithelial cells, intestinal myofibroblasts and monocyte-macrophage cells can be efficiently treated with inflammatory cytokines to induce inflammatory responses. Caco2BBE (A–C), CCD-18Co (D–F) and THP-1 (G–I) cell lines were treated with three different cytokines to induce pro-inflammatory responses. Cells were harvested at different time points to monitor the kinetics of mRNA production of different genes. Values on Y-axis represent mRNA expression levels relative to ß-actin. Columns represent the mean values of three measurements within a single, representative experiment. Error bars represent SD. (TIF) [file pone.0043361.s002.tif]

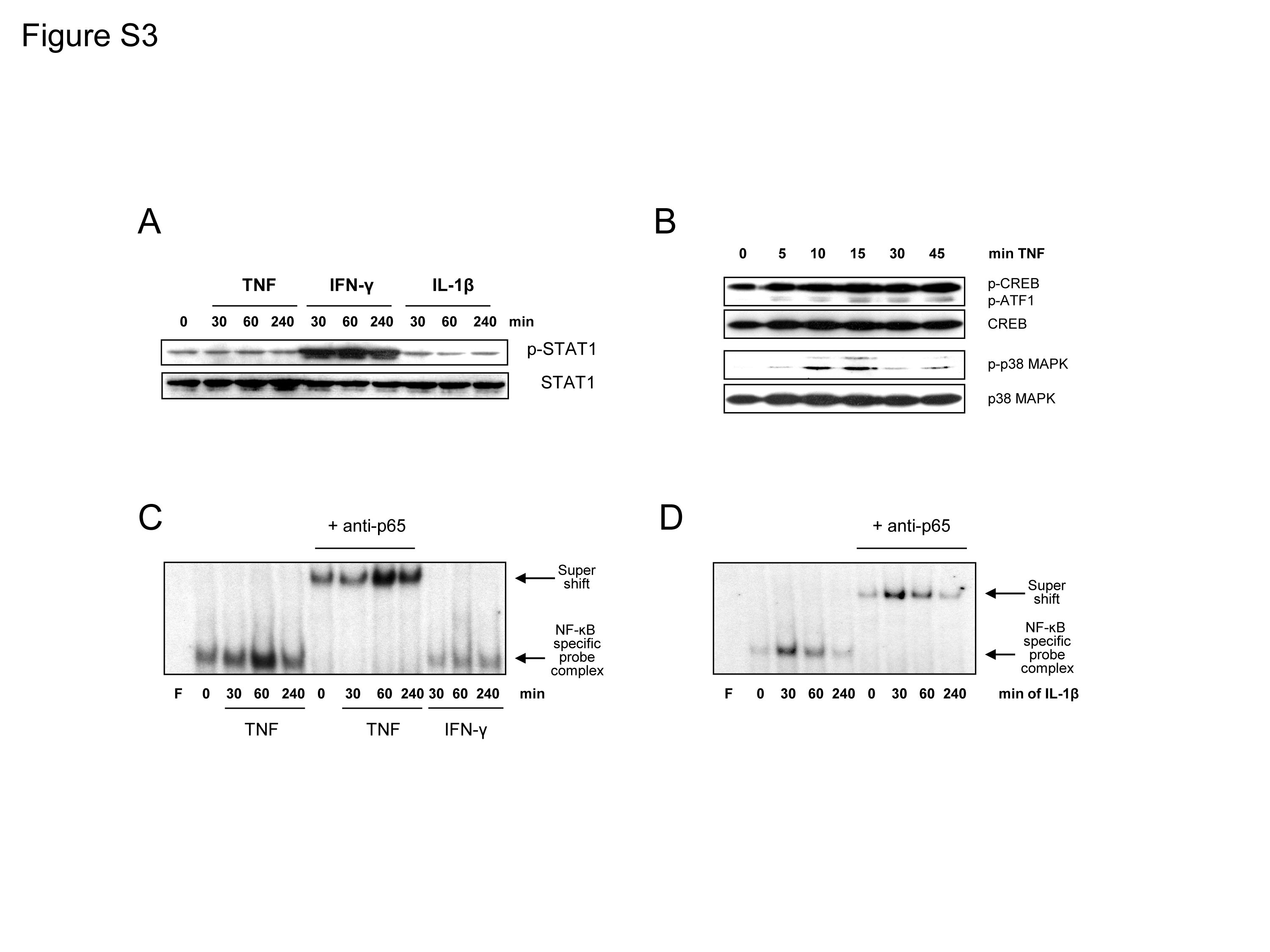

Supplement: Figure S3 — Pro-inflammatory cytokines trigger specific signaling pathways in the cell lines used in the study. (A) IFN-γ, but neither TNF nor IL-1β, induces phosphorylation of STAT1 in intestinal epithelial Caco2BBE cells. (B) TNF induces phosphorylation of CREB, ATF-1 and p38 MAPK proteins in intestine-derived fibroblasts CCD-18Co. (C) TNF, but not IFN-γ, activates NF-κB in Caco2BBE cells as measured by electrophoretic mobility shift assay. Cells were treated with two different pro-inflammatory cytokines to test the specificity of the binding to the NF-κB-specific radiolabelled probe. Maximum activation was observed after 60 min. Addition of anti-p65 antibodies shifts the size of the protein-DNA complexes towards higher molecular weight, showing the specificity of the protein binding to the probe. (D) IL-1β activates NF-κB in Caco2BBE cells as measured by EMSA. Maximum activation was observed after 30 min. All cytokines were used at the concentration of 50 ng/ml. (TIF) [file pone.0043361.s003.tif]

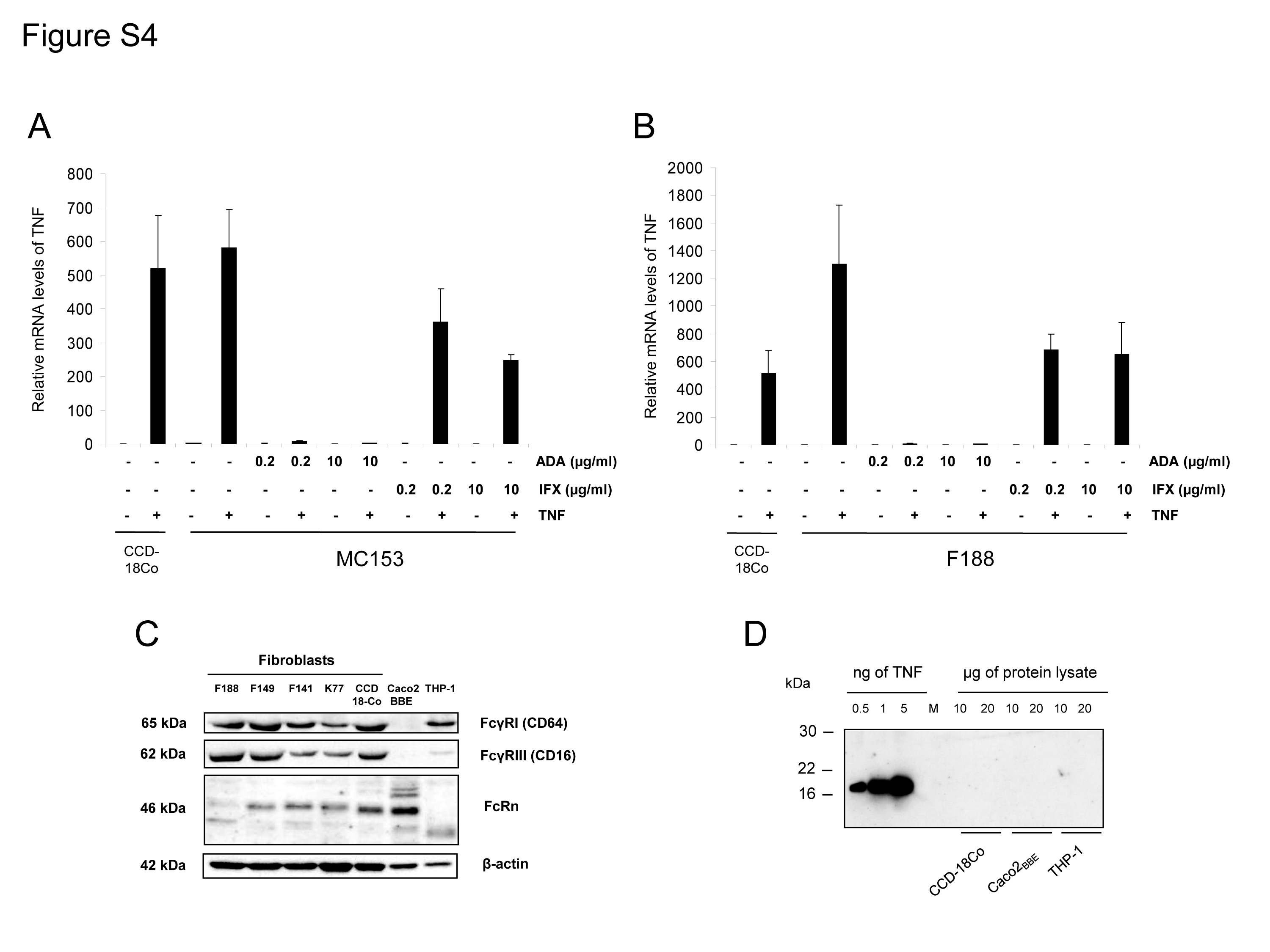

Supplement: Figure S4 — Infliximab has limited efficacy in fibroblasts isolated from CD patients. (A) Fibroblasts isolated from CD patient (MC153) and (B) isolated from fistulizing CD patient (F188) were incubated with either adalimumab or infliximab before treatment with TNF. Columns represent the mean values of three measurements within a single, representative experiment relative to ß-actin. Error bars represent SD. Caco2BBE cells, intestinal fibroblasts and THP-1 cells express Fc receptors (C), but not mTNF (D). Recombinant TNF was used as a positive control (17 kDa). M: Molecular weight marker. (TIF) [file pone.0043361.s004.tif]

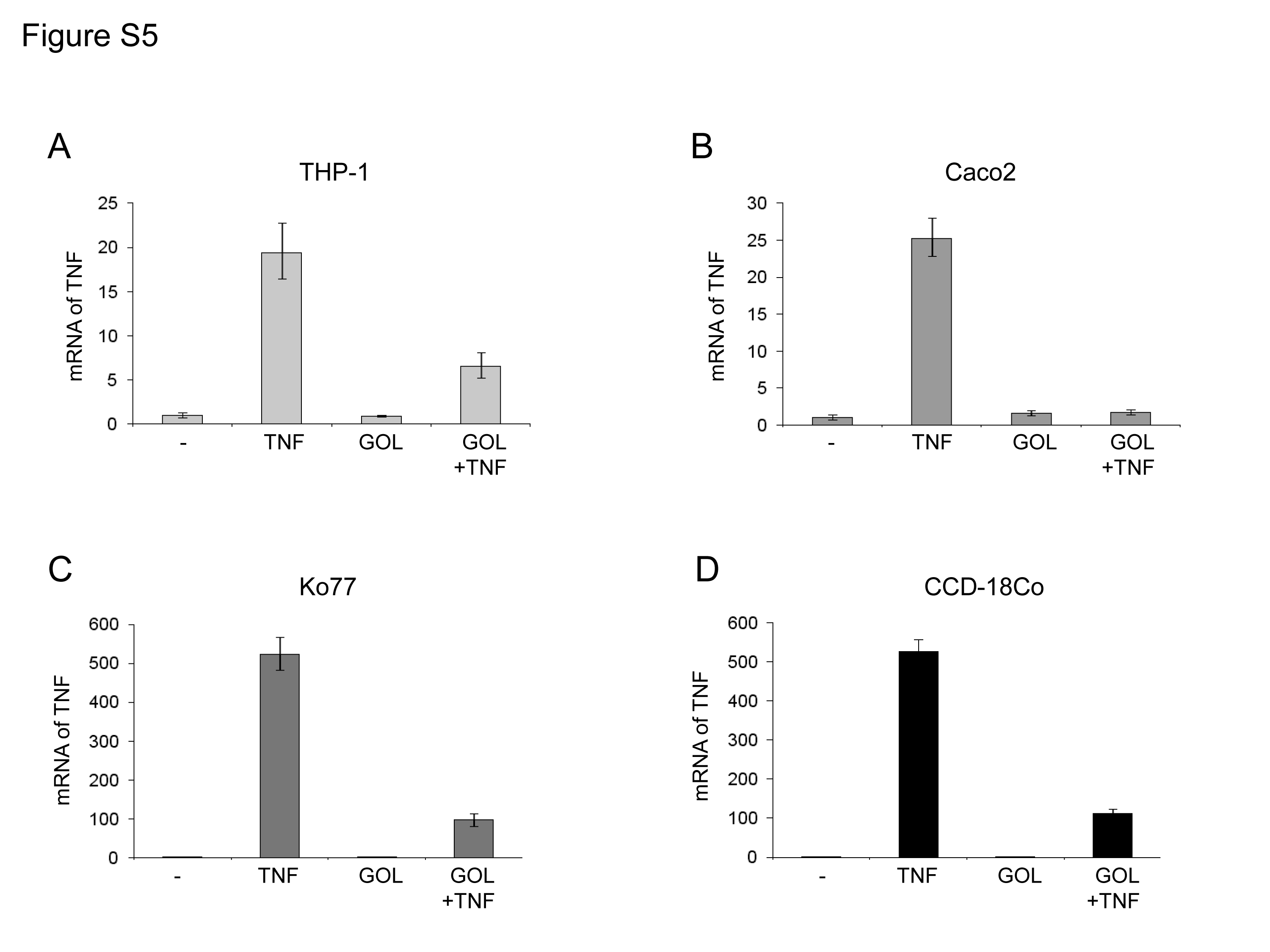

Supplement: Figure S5 — Golimumab displays reduced inhibitory efficacies in intestinal fibroblasts and THP-1 cells, but not in intestinal epithelial Caco2 BBE cell line. (A) THP-1 cells (B) Caco2BBE, (C) Ko77, and (D) CCD-18Co cells were pre-incubated with golimumab and subsequently treated with TNF. The graphs show the results of a single experiment, measured in triplicates. Error bars represent SD. (TIF) [file pone.0043361.s005.tif]

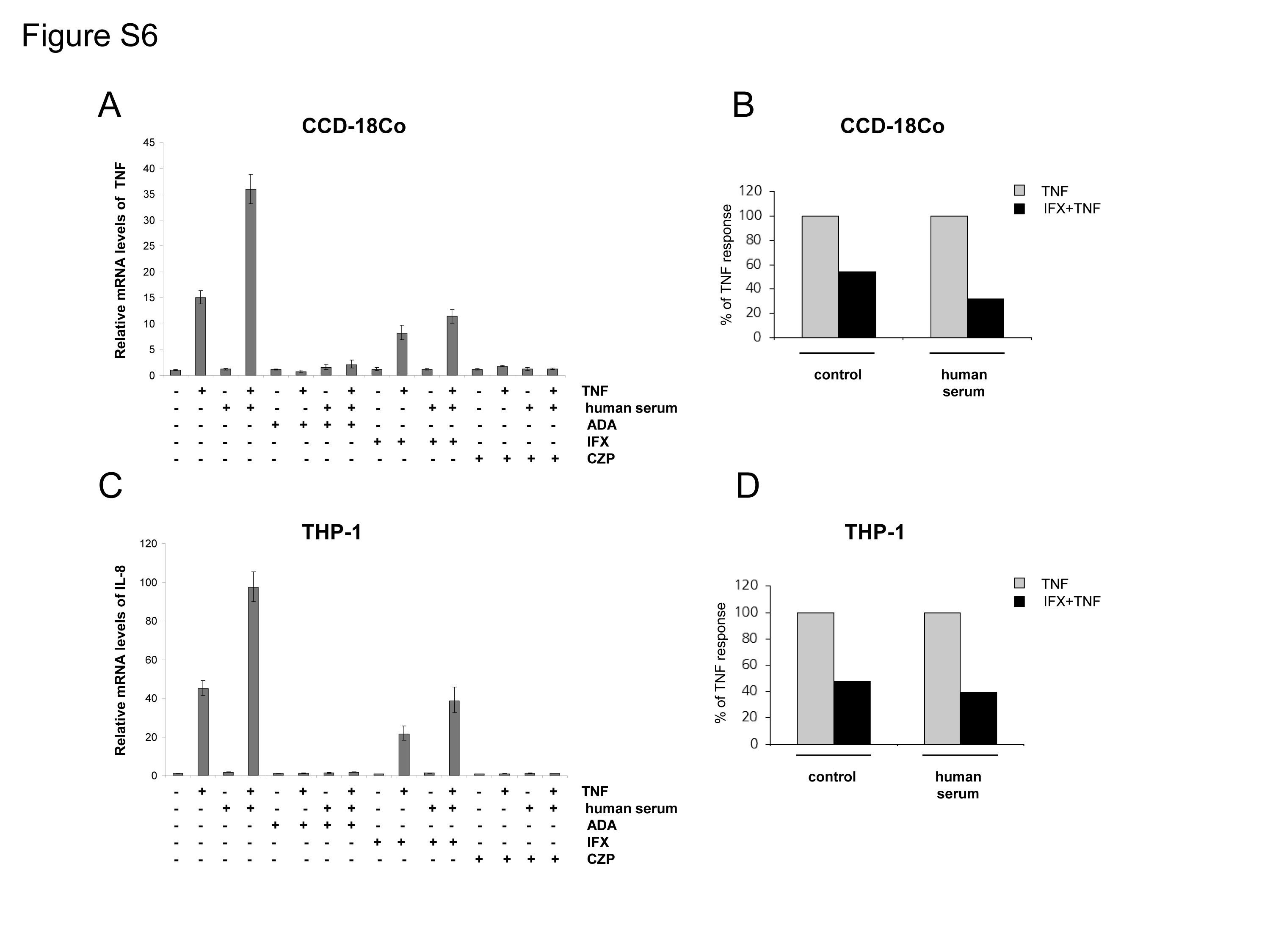

Supplement: Figure S6 — Human serum changes the efficacy of IFX on fibroblasts and monocytes. (A) CCD-18Co fibroblasts were pre-incubated with human serum for 30 min prior to treatment with anti-TNF therapeutics and TNF. Graph shows result of a single experiment measured in triplicate. Error bars indicate SD of three measurements. (B) Quantification of TNF inhibition by infliximab in CCD-18Co fibroblasts expressed as percentage. (C) THP-1 cells were pre-incubated with human serum for 30 min prior to treatment with anti-TNF therapeutics and TNF. Graph shows result of single experiment measured in triplicates. Error bars indicate SD of three measurements. (D) Quantification of TNF-induced response inhibition by infliximab in THP-1 cells expressed as percentage. Graphs show the results of single experiment measured in triplicates. (TIF) [file pone.0043361.s006.tif]

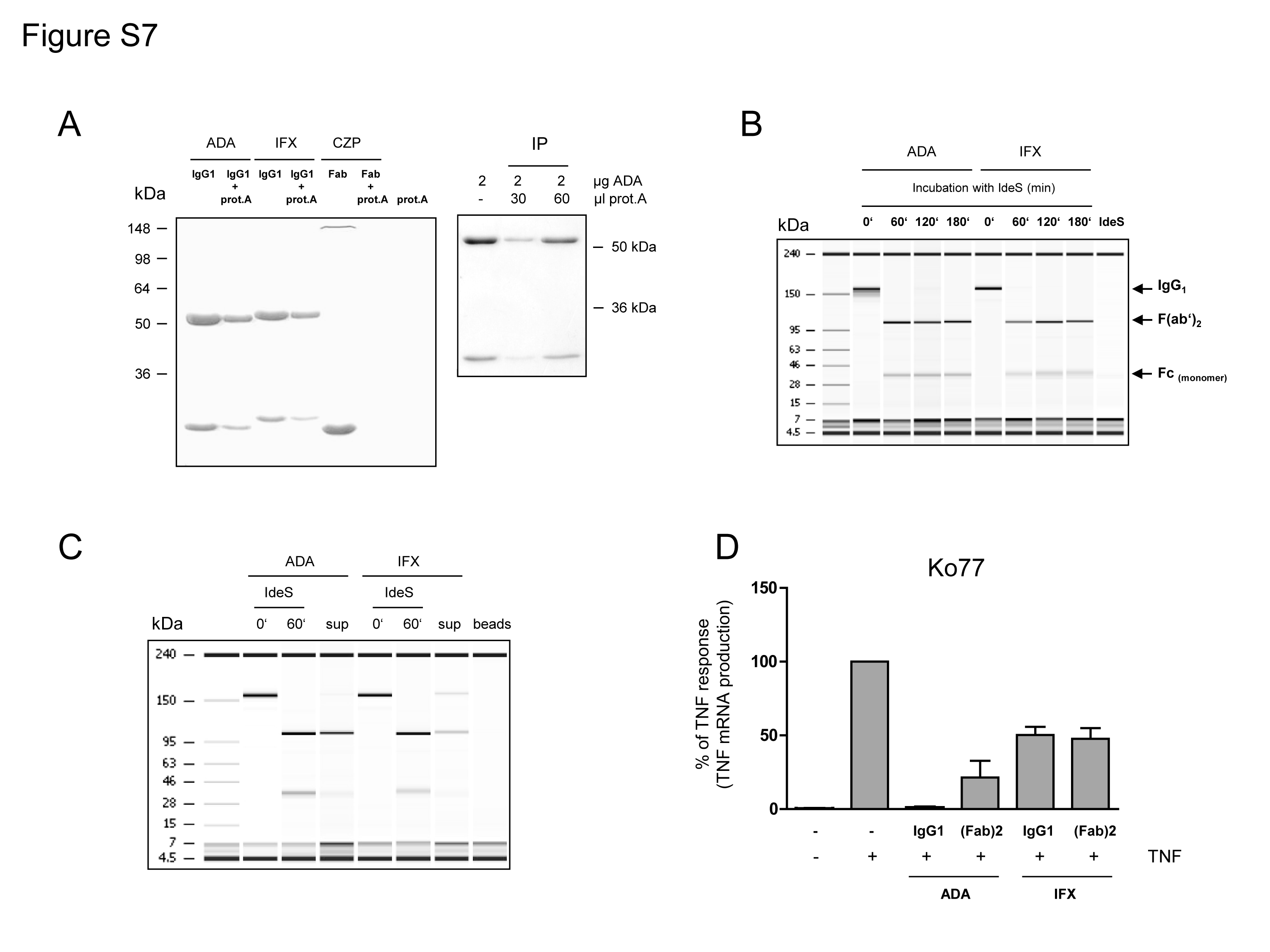

Supplement: Figure S7 — Blocking of Fc and isolation of Fab fragments can be successfully applied for testing the efficacies of anti-TNFs. (A) Both adalimumab and infliximab, but not certolizumab-pegol bind to protein A sepharose beads. (B) Fab and Fc fragments can be produced by incubation with recombinant immunoglobulin-degrading enzyme of Streptococcus (IdeS, FabRICATOR®). (C) After proteolytic digestion Fab fragments of ADA and IFX were removed from the reaction mixture by immunoprecipitation using protein A sepharose beads. (D) Ko77 fibroblasts were pre-treated with either intact IgG1 molecules or Fc-purified digestion mixture containing Fab′ fragments of ADA or IFX and subsequently stimulated with TNF. The percentage of TNF inhibition was assessed based on the production of TNF mRNA by RT-PCR. (TIF) [file pone.0043361.s007.tif]

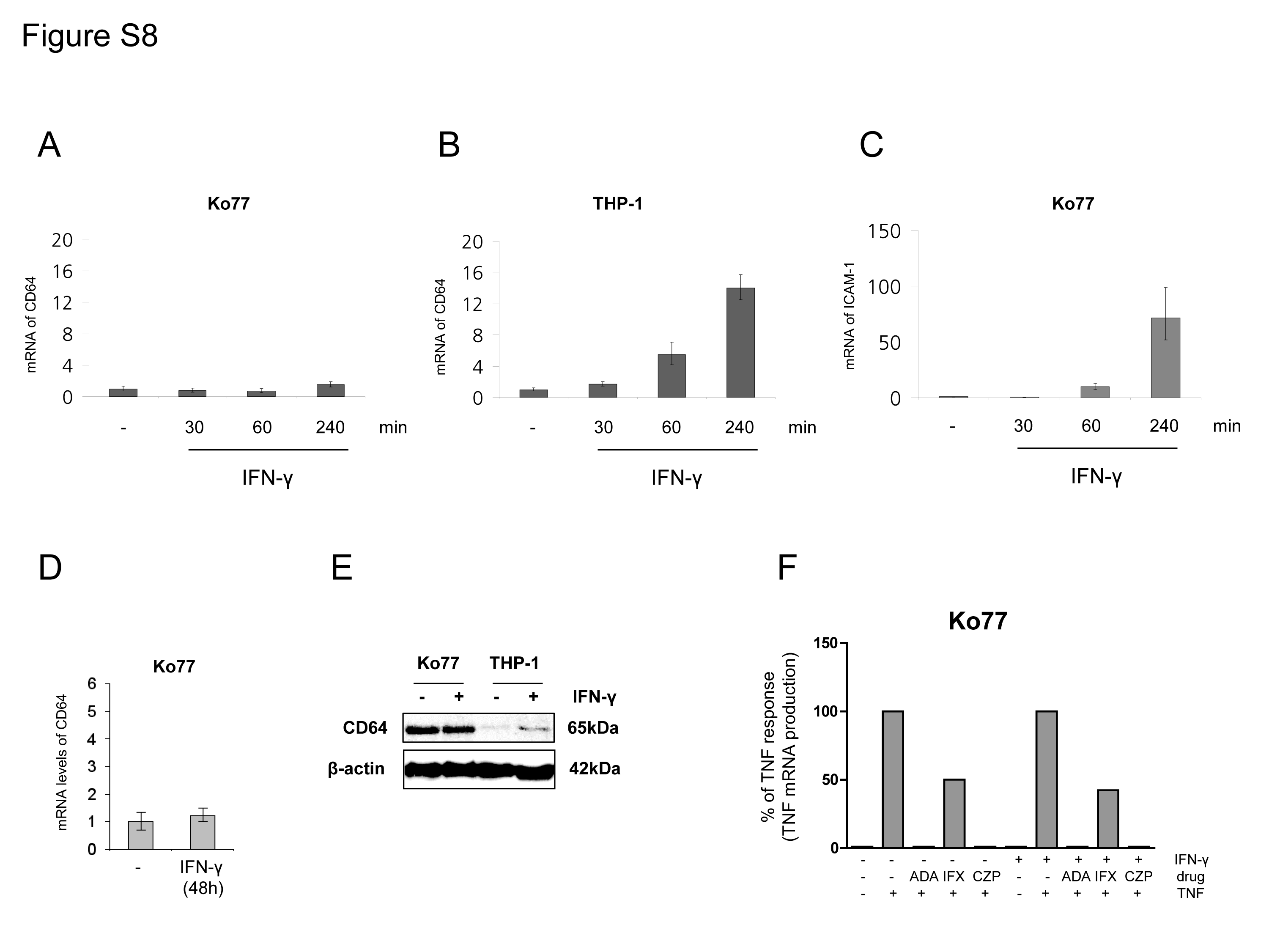

Supplement: Figure S8 — IFN-γ does not induce CD64 mRNA expression and does not influence inhibitory efficacy of IFX in Ko77 fibroblasts. mRNA expression levels of CD64 in Ko77 fibroblasts (A) and THP-1 cells (B) upon IFN-γ treatment. IFN-γ-induced progressive elevation of ICAM-1 mRNA levels in Ko77 (C), which was similar to the effect observed for CCD-18Co fibroblasts (Figure S2,F). (D) mRNA levels of CD64 in Ko77 cells after 48 hours of IFN-γ treatment. (E) Protein levels of CD64 in Ko77 and THP-1 cells after 48 hours of IFN-γ treatment. (F) Pre-incubation with IFN-γ had no impact on inhibitory efficacy of IFX in Ko77 fibroblasts. The graph shows results from a single experiment measured in triplicates. Error bars indicate SD of triplicates. (TIF) [file pone.0043361.s008.tif]

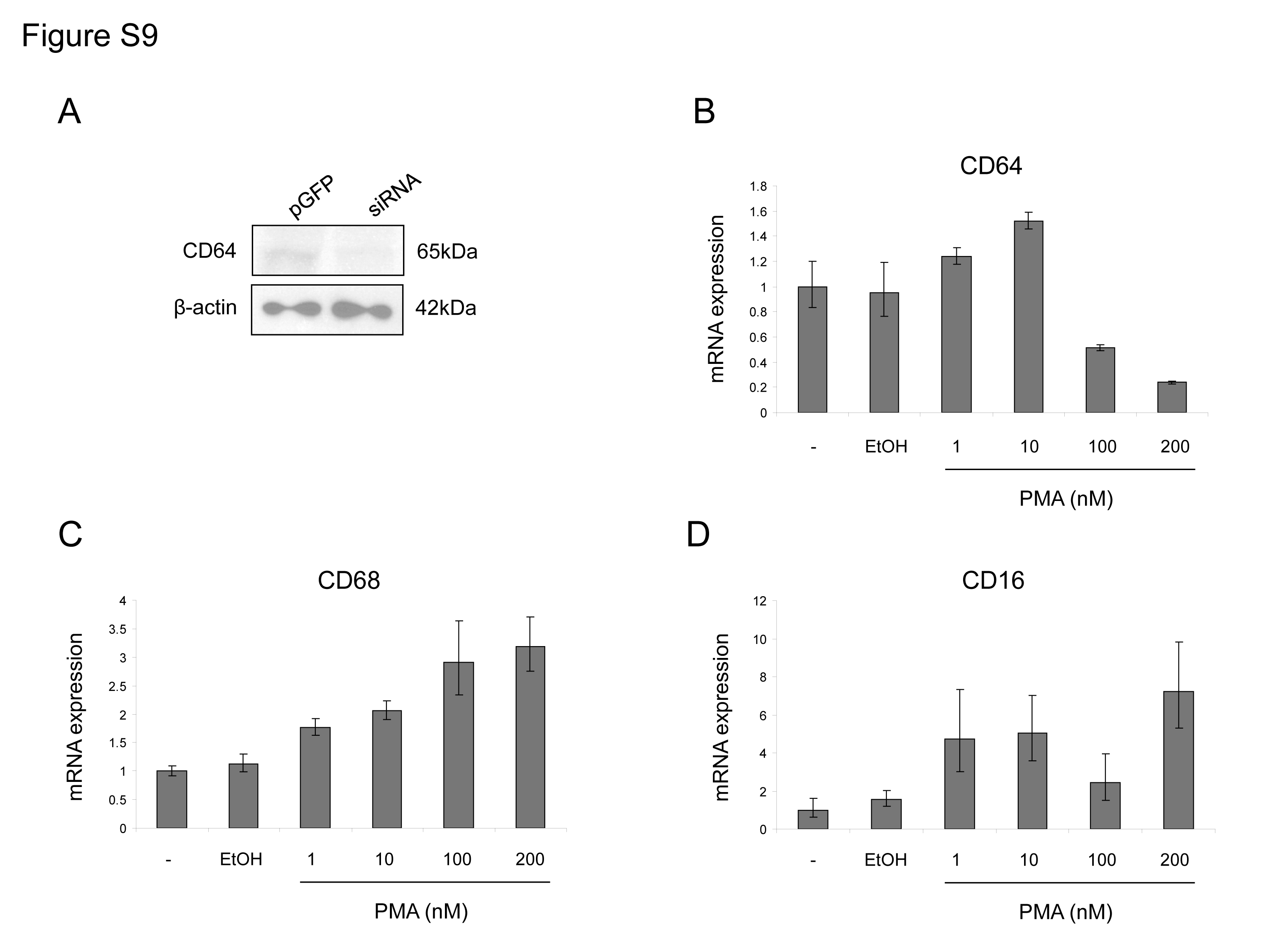

Supplement: Figure S9 — The expression levels of CD64 can be decreased by either siRNA or PMA treatment in THP-1 cells. (A) Western blot analysis of CD64 in pGFP- and siRNA-transfected cells. The mRNA expression levels of CD64 (B), macrophage-specific marker CD68 (C) and CD16 (D) analyzed 48 h after the treatment with different amounts of PMA. The graphs show results from a single representative experiment measured in triplicates. Error bars indicate SD of triplicates. (TIF) [file pone.0043361.s009.tif]

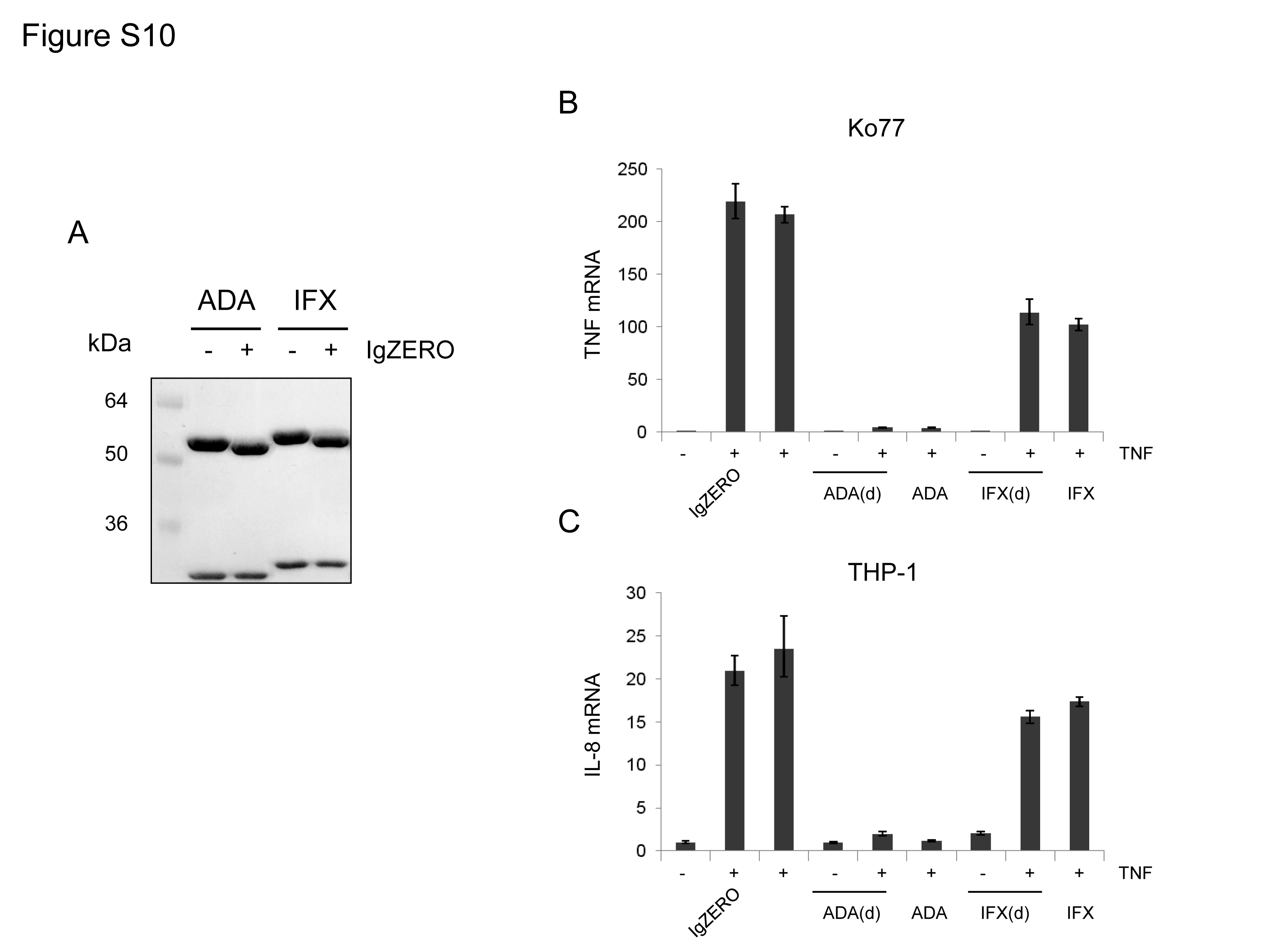

Supplement: Figure S10 — Glycosylation at Asn297 does not influence the inhibitory efficacy of anti-TNF therapeutic antibodies. (A) Both adalimumab and infliximab were deglycosylated at Asn297 as indicated by the shift in molecular mass in SDS-PAGE and Coomassie staining. This modification did not result in significant change in the inhibitory efficacy of anti-TNF antibodies in either Ko77 fibroblasts (B) or in monocytic THP-1 cells (C). The graphs show results from a single experiment measured in triplicate. Error bars indicate SD of triplicates. ADA(d): de-glycosylated adalimumab; IFX(d): de-glycosylated infliximab. (TIF) [file pone.0043361.s010.tif]

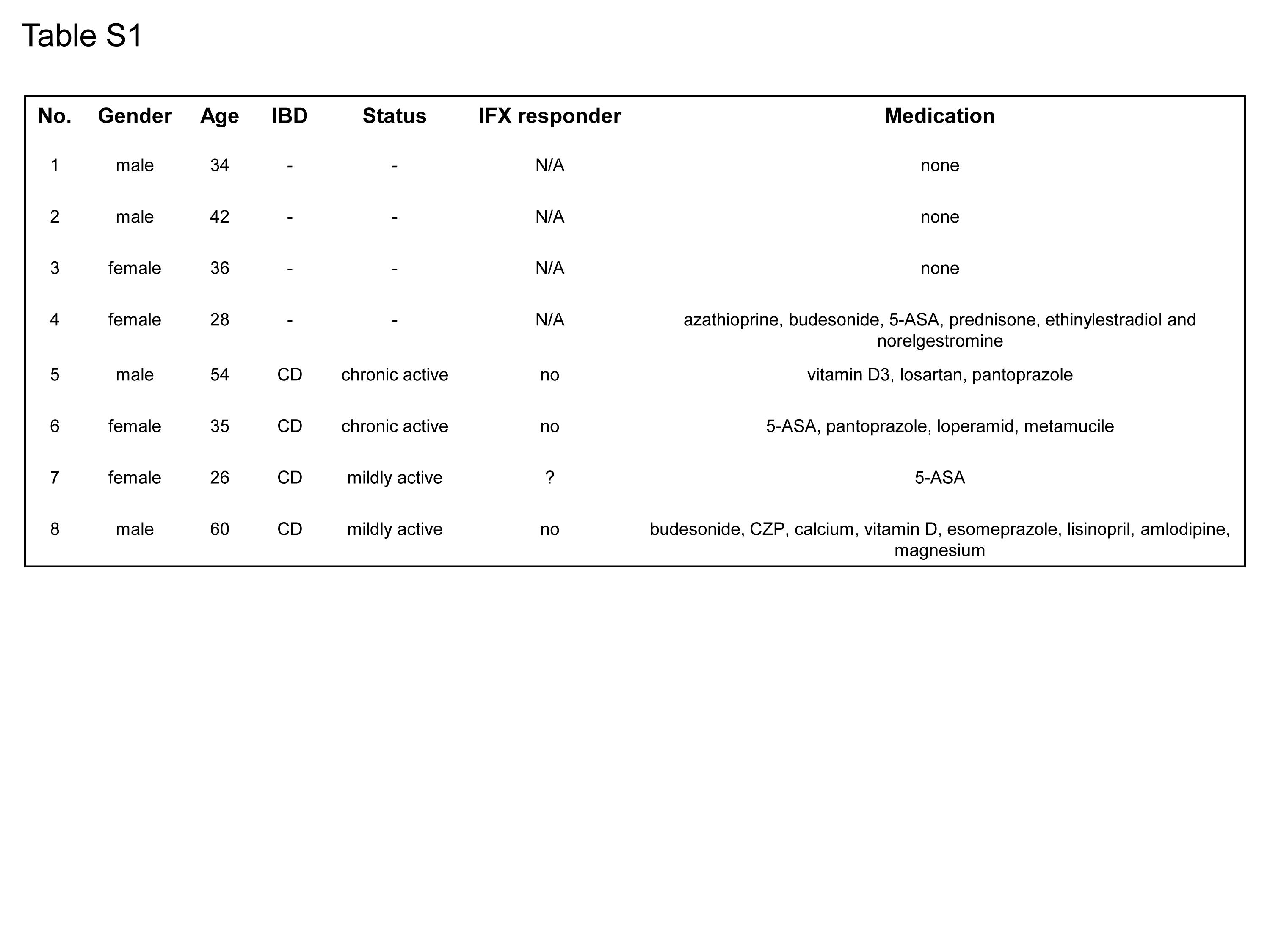

Supplement: Table S1 — Basic characteristics of peripheral blood donors used in the study. 5-ASA: 5-aminosalicylic acid, IFX: infliximab. (TIF) [file pone.0043361.s011.tif]

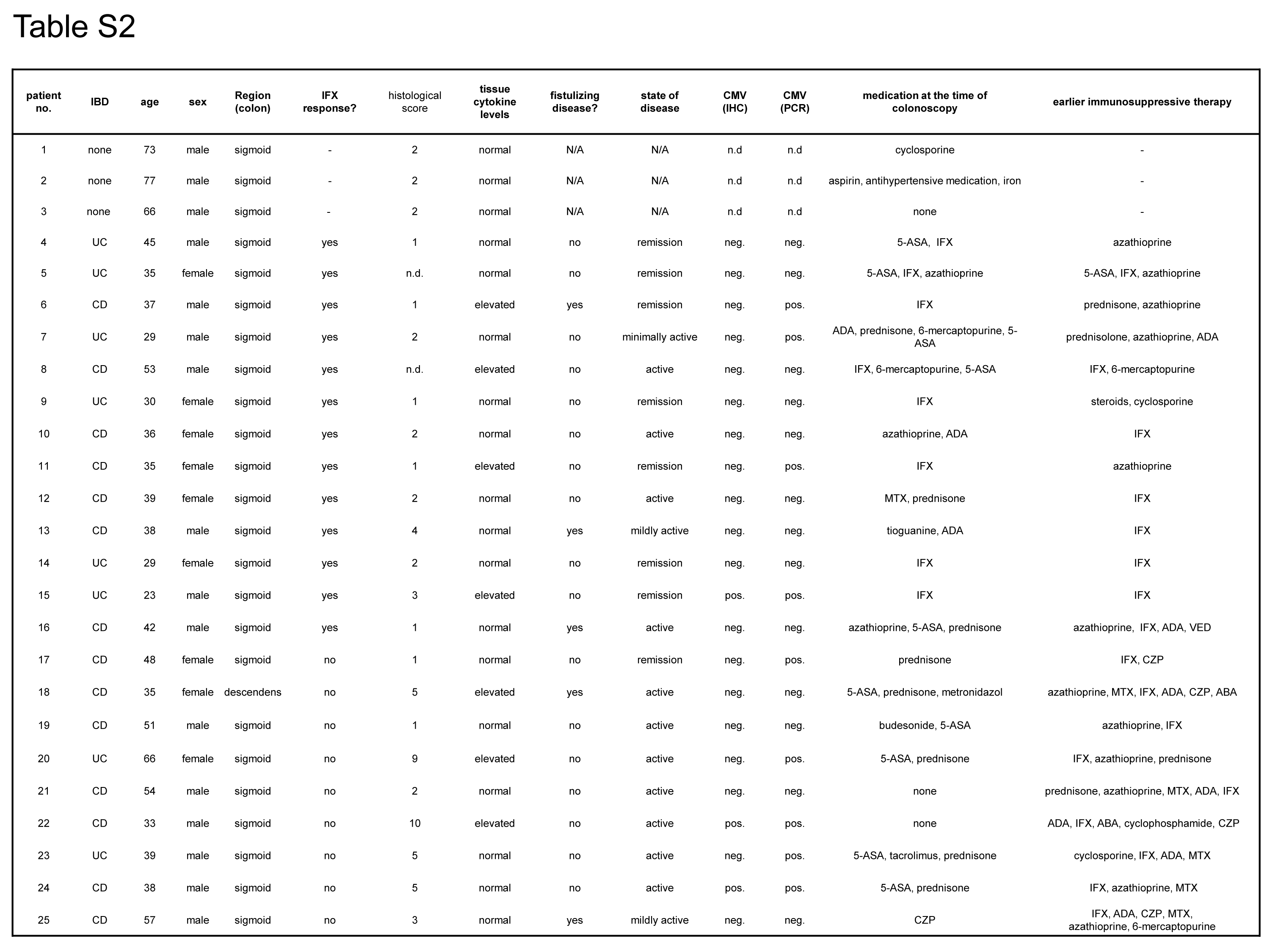

Supplement: Table S2 — Basic description of human intestinal specimens used in the study. 5-ASA: 5-aminosalicylic acid; IFX: infliximab; ADA: adalimumab; ABA: abatacept; VED: vedolizumab; MTX: methotrexate; CZP: certolizumab-pegol; nd: not determined; N/A: not applicable. (TIF) [file pone.0043361.s012.tif]
